# Relevance of porcine intestinal organoids as a surrogate for animal experimentation: application to the investigation of host–virus interactions during porcine coronavirus infection

**DOI:** 10.1186/s13567-025-01657-y

**Published:** 2025-11-21

**Authors:** Ludivine Percevault, Léon-Charles Tranchevent, Lionel Bigault, Maxime Berthaud, Damien Le Gloahec, Pierrick Lucas, Flora Carnet, Aurélie Le Roux, Gérald Le Diguerher, Frédéric Paboeuf, Daniel Dory, Yannick Blanchard, Béatrice Grasland, Maud Contrant

**Affiliations:** 1 Viral Genetics and Biosafety Unit, French Agency for Food, Environmental and Occupational Health and Safety (ANSES), Ploufragan-Plouzané-Niort Laboratory, 41 Rue de Beaucemaine, 22440 Ploufragan, France; 2 UFR of Life Sciences Environment, University of Rennes, 35700 Rennes, France; 3 SPF Pig Production and Experimentation Unit, French Agency for Food, Environmental and Occupational Health and Safety (ANSES), Ploufragan-Plouzané-Niort Laboratory, 41 Rue de Beaucemaine, 22440 Ploufragan, France; 4 Virology, Immunology and Parasitology in Poultry and Rabbits Unit, French Agency for Food, Environmental and Occupational Health and Safety (ANSES), Ploufragan-Plouzané-Niort Laboratory, 41 Rue de Beaucemaine, 22440 Ploufragan, France

**Keywords:** Intestinal organoids, piglet jejunum, transmissible gastroenteritis virus, host–virus interactions, coronavirus, swine, transcriptomic analysis

## Abstract

The small intestine, a part of the digestive system, absorbs nutrients and plays a role in immune protection against viruses that can disrupt its activity. Currently, to analyse these functions, studies rely on in vivo (animals) or in vitro (most often immortalized cell lines) experiments. However, these models have ethical and methodological concerns. Organoids, an ex vivo model, consisting of 3D self-organized cell complexes to recapitulate the cellular diversity, structure, and functionality of an evaluated organ, represent an alternative system. However, to infect organoids, the apical pole containing viral receptors, which are localized in the lumen of the 3D organoids, must be accessible. For this purpose, 2D organoids, corresponding to dissociated and plated 3D organoids, are typically used. This study aimed to characterize the robustness and fidelity of 3D and 2D organoids in terms of gene expression in jejunum tissue and the contribution of 2D organoids in comparison to those of swine testicular cells (ST cells) and piglet jejunums to decipher host–virus interactions with transmissible gastroenteritis virus (TGEV). Our study revealed that our culture and differentiation procedures enabled the production of 3D and 2D organoids that reproduced intestinal epithelial organization with high repeatability. Similar to infected piglets and ST cells, infected 2D organoids expressed genes involved in innate and antiviral immune responses, with a variety of genes activated as a function of viral load. Organoids, which maintain the cellular diversity of the intestinal epithelium, therefore offer the possibility to decipher the host–virus interactions involved in enteric infections.

**Supplementary Information:**

The online version contains supplementary material available at 10.1186/s13567-025-01657-y.

## Introduction

The small intestine is an important organ in mammals that permits nutrient digestion and absorption, hormone secretion, and immune protection against parasites, bacteria, and viruses, which are key functions of this organ [1]. The porcine small intestine is divided into three sections: the duodenum, jejunum, and ileum. Each section is composed of mucosa containing intestinal epithelium and lamina propria, which are composed of the extracellular matrix, fibroblasts, and immune cells such as B and T lymphocytes, natural killer cells, and macrophages [2]. The intestinal epithelium can regenerate because of the ability of stem cells localized in crypts to proliferate and differentiate, with the support of Paneth cells, which are also located in crypts [3]. The multipotency of stem cells allows the production of diverse epithelial cells present in villi, such as enterocytes, goblet cells, and enteroendocrine cells [3].

Animal experiments are typically used to analyse intestinal function and host‒pathogen interactions, with the major advantage that animals exhibit complete physiological complexity [4]. Nonetheless, the utilization of animals for research is regulated and subject to the 3R rule of Refinement, Reduction, and Replacement, formulated by Russel and Burch in 1959 [5]. As an alternative to animal experiments, ex vivo models based on explant culture have been developed to analyse intestinal permeability and absorption [6]. However, these models have severe limitations, such as tissue survival time and interindividual variability [7].

Cell lines represent another experimental system that can be used to analyse intestinal functions such as intestinal barrier function, cellular transport, permeability, and pathogenesis [8]. Some commonly used cell lines derived from the porcine intestinal tract are the intestinal porcine epithelial cell line 1 (IPEC-1), which is derived from the ileum and jejunum, and the intestinal porcine epithelial cell line J2 (IPEC-J2), which originates from the midjejunum of an unsuckled piglet [9]. IPEC-J2 cells have often been used to study pathogen infections caused by rotaviruses, transmissible gastroenteritis virus (TGEV) or bacteria such as *Lactobacillus* [9, 10]. However, a significant proportion of virology studies rely on cell lines that are not natural target cells, such as porcine kidney PK15 cells, which have been used to study astrovirus infection, or LLC PK1 cell lines, which are used to produce porcine deltacoronavirus (PDCoV) [11, 12]. For instance, swine testicular cell lines (ST cells) are used not only to produce and titrate virus but also to analyse the innate immune response against TGEV, which has a tissue tropism for the intestine and lung [13]. Even when they are extracted from relevant intestinal tissues, one of the major limitations of these cell line models is the lack of cellular diversity [14].

Organoids represent an alternative experimental model for counteracting these experimental issues. The first intestinal organoid models were developed in 2009 from the small intestine and colon of mice by Hans Clevers’ laboratory, while the first porcine intestinal organoids from the small intestine and colon were developed in 2013 [15, 16]. Organoids are 3D self-organizing cells that rely on the ability of stem cells to proliferate and differentiate into different cell types to represent the required cellular diversity and to mimic the structure of the epithelium of the organ from which they originate [15]. Porcine intestinal organoids allow the analysis of nutrition, drug efficiency, and host‒pathogen interactions, including those involving parasites, bacteria, and viruses [10, 17–20]. To study host‒pathogen interactions, 2D organoids are often preferred for their access to the apical pole, which is embedded in the inner face of 3D organoids [20].

To study the permissiveness of porcine intestinal organoids to viral infection and their potential added value in studies on host‒pathogen interactions, we used TGEV as a pathogen model. This alphacoronavirus is an enveloped virus containing a 28.5 kb single-stranded RNA genome with positive polarity [21]. It causes anorexia, vomiting, diarrhoea, and up to 100% mortality in piglets less than two weeks old [22]. Its main viral receptor is porcine aminopeptidase N (pAPN), which is expressed by epithelial cells of the intestine and lungs [23, 24]. However, some TGEV strains are also capable of infecting cells that do not express pAPN, suggesting the presence of an additional yet unidentified receptor [23]. A few studies have analysed the activation of the interferon pathway in 2D organoids after TGEV infection [20, 25].

This study aimed to analyse the relevance of 3D and 2D porcine intestinal organoids with several passages from three donor piglets, to characterize the reproducibility of the model and its ability to represent the cellular diversity of the intestinal epithelium, and to summarize the expression of genes involved in intestinal functions. The second part of this study focused on the contribution of 2D organoids to deciphering host–virus interactions, notably the intestinal epithelium barrier and innate and antiviral responses, in comparison with in vitro and in vivo models of TGEV infection. This study aimed to better characterize the advantages and limitations of using 3D and 2D organoids to analyse intestinal functions and host–virus interactions.

## Materials and methods

### Culture of ST cells and virus strains

ST cells (passage 53) were cultivated in 25 cm^2^ flasks in Eagle’s Minimum Essential Medium (EMEM; Lonza, Switzerland), supplemented with 10% FBS (Eurobio, France), 1% penicillin/streptomycin (Thermo Fisher, USA) and 1% GlutaMAX (Thermo Fisher).

The TGEV strain used belongs to the Purdue cluster and is derived from the Purdue P115 strain. The genome sequence is available at the ENA under the accession number PRJEB88925. In comparison with the Purdue P115 strain, this TGEV strain has 29 mutations in its RNA genome sequence (GenBank accession number: DQ811788.1). This virus was produced, passaged and titrated on ST cells.

### 3D and 2D organoid production

#### Isolation and culture of crypts from jejunum tissues

Jejunum tissues were harvested from three stillborn specific pathogen-free (SPF) piglets that were free of TGEV and other enteritic viruses. A five-centimetre section from each tissue was washed with PBS (Sigma, USA) containing 1% penicillin/streptomycin (P/S) (Thermo Fisher) and 7 µg/mL gentamicin (Sigma). This tissue was cut and opened longitudinally to reveal the villi. It was immersed in a solution of PBS (Sigma) containing P/S (Thermo Fisher), gentamycin (Sigma), and 3 mM dithiothreitol (DTT) (Fisher Bioreagent, USA) and scraped to remove the villi under a binocular magnifying glass. After several washes with PBS and antibiotics, the tissue was agitated for 30 min at 80 revolutions per min (rpm) with PBS (Sigma) containing 9 mM ethylenediaminetetraacetic acid (EDTA) (Sigma), 3 mM DTT (Fisher Bioreagents), 10 µM ROCK inhibitor Y27632 (ATCC), 1% P/S (Thermo Fisher), and 7 µg/mL gentamicin (Sigma). Crypts were harvested by scraping into PBS (Sigma) containing 10 µM Y27632 (ATCC), 1% P/S (Thermo Fisher), and 7 µg/mL gentamicin (Sigma). After 200 µm filtration, the crypts were centrifuged at 100 × *g* for 5 min at 4 °C. The supernatants were discarded, and the crypts were washed with DMEM/F12 (Gibco, USA) and 10% foetal bovine serum (FBS) (Eurobio) and counted on a Kova slide. A total of 150 crypts were embedded in 50 µL of Matrigel (Corning, USA) and transferred to 24-well plates. After 20 min of polymerization, 500 µL of 3D organoid growth culture medium (Intesticult Human Organoid Growth Medium (cat. # 06010, Stemcell, Canada), with a 1:1 ratio of basal medium and supplement) supplemented with 10 µM ROCK inhibitor Y27632 (ATCC, USA) was added. After 1 day of culture, the medium was changed to 3D organoid growth culture medium without the ROCK inhibitor Y27632 (ATCC). The remaining crypts were frozen at −80 °C in a mixture of DMEM F12 (Gibco), 10% FBS (Eurobio), and 10% dimethyl sulfoxide (DMSO) at approximately 600 crypts per vial.

#### Maintenance of 3D organoids in culture

The culture medium was changed every 3 days. Every 8 days, the 3D organoids were passaged by adding 1 mL of TRYple Express (Thermo Fisher) to degrade the Matrigel dome (Corning) and dissociating the cells by vigorous pipetting for 10 min at room temperature. The cells were centrifuged again at 300 × *g* for 5 min at 4 °C. The supernatant was removed, and the cells were washed with DMEM F12 (Gibco) supplemented with 10% FBS (Eurobio). The cells were subsequently centrifuged at 300 × *g* for 5 min at 4 °C. Each well of the 3D organoid was diluted four times and transferred to Matrigel (50 µL dome per well of a 24-well plate). After polymerization, 500 µL of 3D organoid growth medium supplemented with 10 µM ROCK inhibitor Y27632 (ATCC) was added and changed after 1 day of culture to remove the ROCK inhibitor Y27632. After 5 and 14 passages, 3D organoids were frozen in DMEM F12 supplemented with 10% FBS (Eurobio) and 10% DMSO (Sigma) at 1 million cells per vial.

#### 2D organoid production

3D organoids (passage 25) were dissociated with TRYple Express (Thermo Fisher). The dissociated organoids were subsequently centrifuged at 300 × *g* for 5 min at 4 °C. The supernatant was removed, and the dissociated organoids were washed with DMEM F12 supplemented with 10% FBS (Eurobio). The dissociated organoids were subsequently centrifuged at 300 × *g* for 5 min at 4 °C. The cells constituting the organoids were counted by seeding 100 000 cells per well in 24-well plates. The necessary quantity of cells was centrifuged at 300 × *g* for 5 min at 4 °C and suspended in 2D organoid growth medium (Intesticult Human Organoid Growth Medium (Stemcell) (at a 1:0.8 ratio of basal medium and supplement)). Five hundred microlitres of suspended cells were placed in each well of 24-well plates coated with 0.5% Matrigel (Corning).

### Infection of ST cells and 2D organoids

For infection, ST cells were trypsinized and cultivated in 6-well plates at 500 000 cells per well in triplicate in EMEM (cat. # 12-662Q, Lonza) containing 10% FBS, 1% P/S (Thermo Fisher), and 1% GlutaMAX (Thermo Fisher). After 1 day of culture at 37 °C and 5% CO_2_ and two washes with EMEM without FBS and supplemented with 1% P/S (Thermo Fisher), ST cells (approximately 70% confluence) were inoculated with TGEV for one h at an MOI of 0.06. The control conditions involved mock infection with the ST cell supernatant. Two washes were performed, and 2 mL of EMEM (Lonza) supplemented with 1% P/S (Thermo Fisher) was added.

After 3 days of culture, the 2D organoid cell confluence was close to 70%, and the monolayer was washed twice with DMEM F12 and inoculated for one h at 37 °C and 5% CO_2_ at an MOI of 0.06 or an MOI of 1 with the TGEV strain in quadruplicate per donor piglet. Control cells were mock-infected with ST cell supernatant. After 1 h of inoculation, two washes were performed with DMEM F12, and 500 µL of 2D organoid growth medium was added to each well.

For the ST cells and 2D organoids, the supernatant and cells were collected at 0, 3, 6, 9, 16, 24, and 48 h post-infection (hpi). To collect 2D organoid cells, 500 µL of TRYple Express (Gibco) was added to the cell monolayer to collect the cells in 1.5 mL Eppendorf tubes. After centrifugation at 300 × *g* and 4 °C for 5 min, the supernatant was removed, and 1 mL of TRIzol Reagent (Thermo Fisher) was added to the pellet. For the ST cells, 1 mL of TRIzol reagent was added directly to the monolayer. The supernatants and cell pellets were lysed in TRIzol reagent and stored at −80 °C until RNA extraction.

### Immunostaining of 3D and 2D organoids

#### Preparation and fixation of 3D and 2D organoids

At the 25^th^ passage, the medium of the 3D organoids was removed, and the organoids were washed twice with PBS. The 2D organoids were produced, as described above, in 96-well plates, with 10 000 cells per well. After 3 days of culture, the medium was removed, and the monolayers were washed three times with PBS, followed by the addition of 350 µL or 200 µL of 4% paraformaldehyde (PFA) (Biotium, USA) for 30 min at room temperature for 3D or 2D organoids, respectively. After fixation, to avoid destruction of the 3D structures, the tip of a 1000 µL cone was cut with a sterile scalpel to widen the mouth. The 3D organoids were harvested and transferred to Eppendorf tubes. After 5 min of sedimentation, the supernatant was removed, and the 3D organoids were washed with PBS.

#### Permeabilization and incubation with antibodies

After being washed once with PBS, 150 µL or 300 µL of PBS supplemented with 5% BSA (Eurobio) and 0.4% Triton (Sigma) was added to the 3D or 2D organoids. This solution was incubated overnight for the 3D organoids or for 1 h at room temperature for the 2D organoids. Following two washes, primary antibodies (150 µL for 3D organoids or 100 µL for 2D organoids) targeting zonula occludens 1 (ZO1), lysozyme (Lyz), leucine-rich repeat-containing G-protein-coupled receptor 5 (Lgr5) and villin 1 (VIL1), diluted in 0.1% BSA in PBS (see Table 1 for volume and dilutions performed) were incubated for 2 or 1 h at room temperature for the 3D organoids or 2D organoids, respectively. After two washes with 0.1% BSA in PBS, 150 µL or 100 µL of secondary antibodies (targeting mouse and rabbit antibodies) diluted in 0.1% BSA in PBS was incubated for 2 or 1 h at room temperature for 3D organoids or 2D organoids, respectively (see Table 2 for volume and dilutions). After two washes with 0.1% BSA in PBS, the DNA was labelled with DRAQ5 (BioLegend, USA) diluted 1:800 for the 3D organoids. The DNA of the 2D organoids was labelled with 1 µg/mL Hoechst (Sigma) for 10 min at room temperature. The fluorescent signals of VIL1, ZO1 and Lyz in the 3D and 2D organoids were imaged with Operetta CLS (Revvity, USA) with Harmony SW (Revvity) software. The staining of pAPN, viral N protein and Lgr5 in 2D organoids was imaged with an Olympus CKX41. The sample preparation and image post-processing procedures were, however, the same for all the imaged proteins. Merged images were generated with Gimp software (v2.10.8).

**Table 1:** **Primary antibodies used in this study**

Protein target	Reference	Dilution	Species
Aminopeptidase N	Homemade antibody given by VIM unit INRAE lab	1:500	Mouse
Lysozyme	PA5-16668-USA-Invitrogen	1:100	Rabbit
Villin 1	1D2C3-sc-58897-USA-Santa Cruz	1:300	Mouse
Lgr5	PA5-23000- USA-Invitrogen	1:100	Rabbit
ZO1	R40-76, sc-33725-USA-Santa Cruz	1:100	Rat
Actin	A5441, USA-Sigma	1:100	Mouse
Viral N protein	Homemade antibody given by Pirbright Institute	1:1000	Mouse

**Table 2:** **Secondary antibodies used in this study**

Species target	Reference	Dilution	Fluorochrome
Rabbit	35553-Thermo Fisher	1:300	Alexa Fluor 488
Mouse (used to target rat antibody)	A-11001-Invitrogen	1:300	Alexa Fluor 488

### Animal experiments

An ethics committee and the French Ministry of Higher Education and Research approved all the experimental procedures (authorization number: #39769–2022120716251321). SPF piglets free of TGEV were oro-nasally inoculated with 7 mL of EMEM (2 mL nasally and 5 mL orally) for the control piglets or with 10^4.75^ TCID_50_ per piglet, corresponding to 10^8^ copies of the viral N gene per infected piglet (*n* = 8). Each experimental group of piglets was housed in a separate room in an animal biosafety level 3 (ABSL3) facility. Faecal and nasal swabs and blood samples were collected on Days 0, 1, and 2 after inoculation. At 24 hpi (*n* = 4 per group) and 48 hpi (*n* = 4 per group), different organs from the piglets, including the jejunum, duodenum, ileum, colon, lung, trachea, bronchi, trachea-bronchial, and mesenteric lymph nodes, were collected and placed in RNAlater (Thermo Fisher) to preserve RNA quality.

### RNA extraction

RNA was extracted with either TRIzol Reagent (Thermo Fisher) for piglet tissues, crypts, organoids (2D and 3D) and ST cells or TRIzol LS (Thermo Fisher) for cell culture supernatants according to the supplier’s recommendation for all experimental models. The piglet jejunum tissues stored in RNAlater (Thermo Fisher) were transferred into Eppendorf tubes with 1 mL of TRIzol Reagent (Thermo Fisher) and crushed with a shredder.

Crypts, 3D and 2D organoids and ST cells were lysed and frozen (−80 °C) in 1 mL of TRIzol until extraction.

After extraction, the RNA was treated with a TurboDnase-free kit (Thermo Fisher) and quantified with a Quant-it RNA kit (Thermo Fisher). RNA quality was checked with a Fragment Analyzer (Agilent, USA). RNA from 100 µL of supernatant from ST cells and 2D organoids, previously frozen at -80 °C, was extracted with TRIzol LS (Thermo Fisher) following the supplier’s instructions.

### Reverse transcriptase quantitative PCR (RT‒qPCR)

To assess the viral N gene, RNA from 100 µL of the supernatant was recovered in 50 µL of water, and 5 µL of this RNA preparation was used for RT‒qPCR analysis. Four nanograms of cellular RNA per well was amplified by RT‒qPCR following the manufacturer’s instructions. The assessment of glyceraldehyde-3-phosphate dehydrogenase (GAPDH) and hypoxanthine phosphoribosyltransferase 1 (HPRT1) was achieved with a Luna Universal One Step RT qPCR kit (NEB, USA). Kit optimization was achieved with 1X Luna WarmStart RT Enzyme Mix and 0.5X Luna Universal One-Step. A TaqMan RNA-to-CT one-step kit (USA, Applied Biosystems) was used for assessment of the viral N gene. A standard curve ranging from 10^9^ to 10^2^ copies of tenfold serial dilutions of viral N gene RNA (943 bp in length) produced in vitro was used to quantify the number of viral N gene copies per sample. Controls without the RT enzyme were also used to demonstrate the absence of DNA contamination. The primers and probes used for this study are listed in Tables 3 and 4. The relative expression levels of cellular RNA were determined on the basis of the cycle threshold (∆∆^CT^) method, normalized to expression of GAPDH and HPRT1, and compared with the 0 h time point. The average of the ∆∆^CT^ results of the two housekeeping genes for each time point was calculated.

**Table 3:** **Primers used in this study**

Gene name	Reverse primer	Forward primer	References
TGEV (N gene)	ACATTCAGCCAGTTGTGGGTAA	GCAGGTAAAGGTGATGTGACAA	[77]
GAPDH	ATGACAAGCTTCCCGTTCTC	ACCTCCACTACATGGTCTACA	[78]
HPRT1	GGACTTGAATCATGTTTGTG	CAGATGTTTCCAAACTCAAC	[79]

**Table 4:** **Probe used in this study**

Gene name	Probe sequence	References
TGEV (N gene)	6 FAM TGG CAC TGC TGG GAT TGG CAA CGA MGB	[77]

### RNA sequencing (RNA-seq)

For RNA-seq analysis, 9 hpi and 24 hpi were chosen for the analysis of the kinetics of the ST cells (MOI of 0.06) and 2D organoids (MOI of 0.06 and MOI of 1). Three replicates of the ST cells were analysed. Four replicates of each 2D organoid donor pig (*n* = 3) were pooled, with equivalent RNA concentrations (125 ng). For the piglet jejunums (in vivo), the 24 hpi time point was selected to analyse the beginning of infection. Three of the four piglet jejunums (in vivo) per infection and control condition were chosen according to the quality of their RNA, as determined by Fragment Analyzer analysis. The RNA was sequenced on a Novaseq600, which was performed by Novogene, and on a NovaSeq X at the iGenSeq core facility at ICM. Libraries were prepared with a Novogene NGS RNA Library Prep Set kit (Novogene, China) and an Illumina Stranded mRNA kit (Illumina, USA), respectively. The dataset generated for this study is available on ArrayExpress under the accession number E-MTAB-15113.

### Analysis of RNA-seq data

RNA sequencing data were processed and cleaned with fastp ([26], v0.23.2). The reads were mapped to the pig transcriptome (v11.1 of the Swine Genome Sequencing Consortium) and counted with Salmon ([27], v0.14.1) and RASflow ([28], v2020). The fragment per kilobase million (FPKM) values were calculated, converted to base 2 logarithm and filtered (> 1) with the edgeR package in R ([29], v4.0.9). The variability in transcript expression between biological replicates and experimental models was measured with the determination coefficient (R^2^), which reflects the quality of the prediction of linear regression. To facilitate the visualisation of the value of FPKM by heatmap representation, the log base e of FPKM + 1 was performed. The normalized read counts for each gene were calculated with the DESeq2 R package ([30], v1.42.0) and converted to logarithm base 2 to facilitate histogram visualization. Differential expression analysis was achieved with DESeq2. Through the use of read counts, principal component analysis (PCA) was performed with the plotPCA function of DESeq2 (v1.42.0) to visualize the difference in gene expression between the models. For the control conditions, the differential gene expression of 3D and 2D organoids compared with that of piglet jejunums was calculated. Under infection conditions, differential gene expression levels were computed and compared to those under the corresponding control conditions. The results were filtered with a cut-off of padj < 0.05 and a log_2_-fold change > 1. The network of genes involved in the extracellular matrix was constructed with STRING (v12.0). Only the main component of the network was further analysed by considering the log_2_-fold changes in 3D and 2D organoids compared to those in the piglet jejunums with Cytoscape software (v3.10.1). The Gene Ontology annotations were retrieved via R (v2023.06.1) and the clusterProfiler package ([31], v4.10.0). The analyses were first performed with pig annotations, but a lack of annotations was observed (Additional file 1). We then compared the number of annotated proteins among swine, mouse and human annotations for a handful of Gene Ontology (GO) terms corresponding to functions expected to be perturbed in our dataset, such as the innate immune response, defence response to virus and intestinal development (Additional file 1). As expected, this revealed that the swine annotations were rather rare and were mostly based on automatic inference. This also revealed a greater number of annotations in mice than in humans. We therefore decided to use mouse annotations by retrieving pig–mouse orthologous genes from the ENSEMBL Biomart website (v111). To confirm our key results, major enrichment analyses obtained with mouse data were reproduced with human annotations.

### Statistical analysis

All the statistical analyses regarding the kinetics of the infection, pAPN expression and expression of genes involved in the NOTCH pathway were performed with Prism (GraphPad Software, Inc., v10.2.3). Unless otherwise stated, the data are presented as the means with standard deviations. To compare the different time points, Mann‒Whitney tests or Kruskal‒Wallis tests were used when two or more than two time points were included in the comparison. pAPN and NOTCH pathway expression in the infected and control conditions was directly compared with an unpaired Student’s *t* test and ANOVA if more than two groups were compared. Differences were considered significant when the *P* value was < 0.05. In the figures, the *P* values are indicated as follows: *P < 0.05; ** *P* < 0.01; *** *P* < 0.001; and **** *P* < 0.0001.

## Results

### Validation of the 3D and 2D organoid models

#### Morphology of the 3D organoids

**Figure 1:**
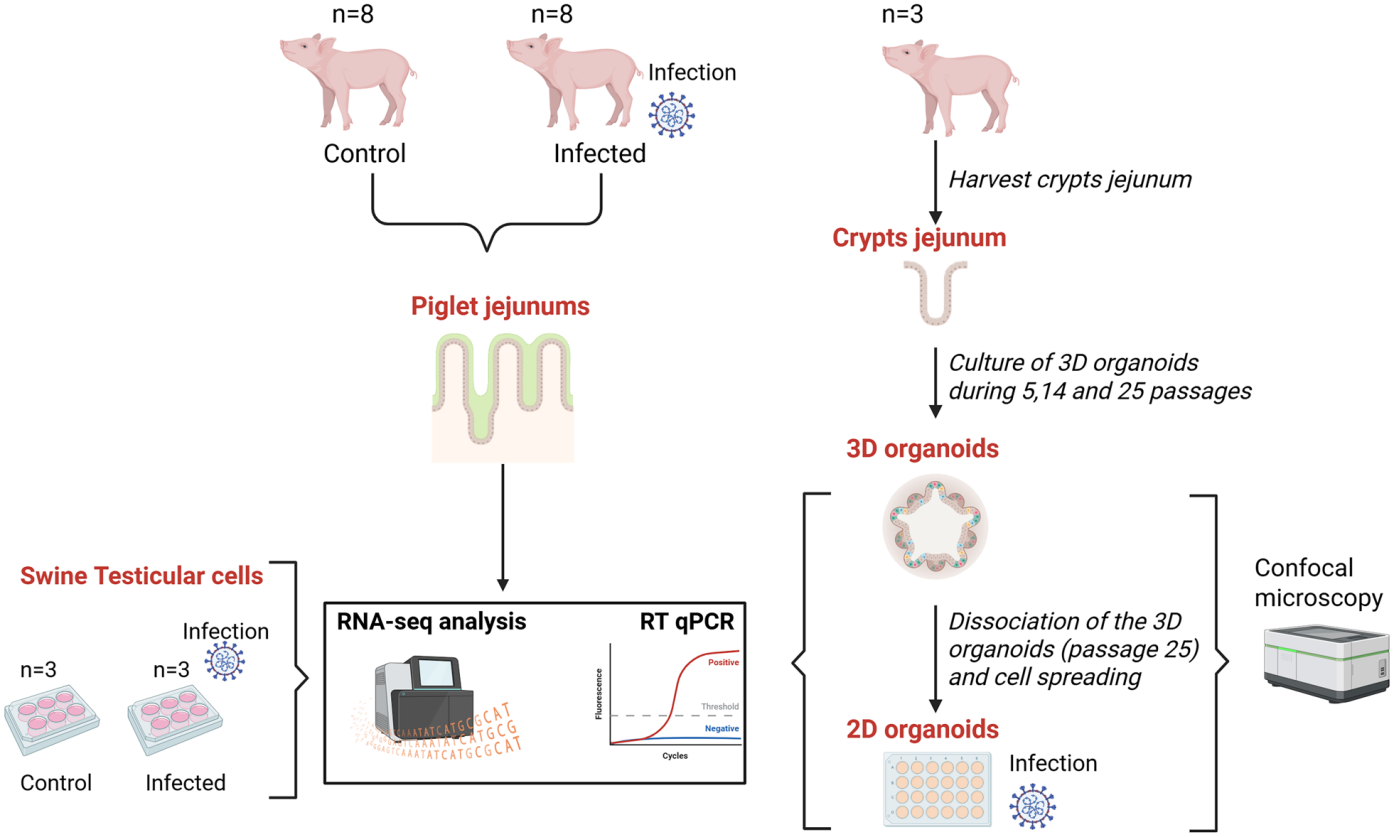
**Graphic representation of the experimental protocol**. For the in vitro model (cell lines), ST cells (passage 53) were cultivated under control and TGEV-infected conditions (three replicates, an MOI of 0.06). For the in vivo model, eight 5-week-old piglets per group were inoculated with EMEM as the control or with 10^4.75^ TCID_50_ as the infection condition. At 24 hpi and 48 hpi, the jejunum from four piglets per group and time point was harvested after euthanasia and necropsy. To produce intestinal organoids, the intestinal crypts of three stillborn piglets were isolated and cultivated to produce 3D organoids. Every week, the cells were dissociated and diluted 1:4 to generate new organoids. Several culture passages (5, 14, and 25) were harvested. After 25 passages, the 3D organoids were dissociated and spread out in two dimensions (2D). The 2D organoids were infected with TGEV at an MOI of 0.06 or an MOI of 1. RNA from piglet jejunums, crypts, and 3D and 2D organoids under control and infection conditions was extracted and analysed by RT‒qPCR (to target the viral N gene) and by RNA-seq. First, the control conditions were compared with RNA-seq (for ST cells, piglet jejunums, crypts, 3D organoids, and 2D organoids). In addition, cellular diversity and polarization were investigated in the organoids through confocal microscopy. In the second part, the infection conditions were compared (for ST cells, piglet jejunums and 2D organoids) via RNA-seq and RT‒qPCR analyses. This graphic representation was designed with Biorender.

**Figure 2:**
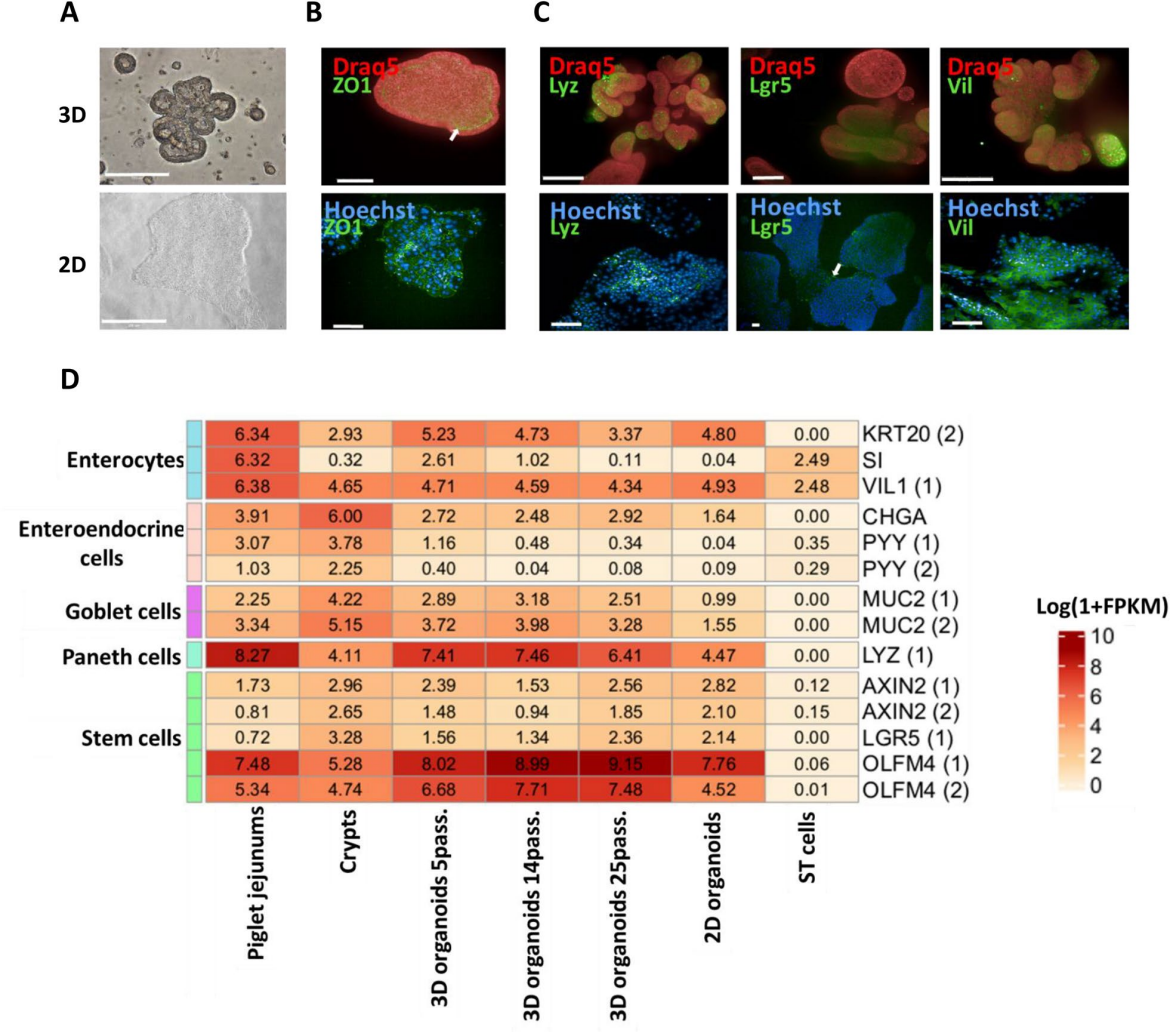
**Comparison of the cellular diversity of 3D and 2D organoids with that of piglet jejunums, crypts and ST cells**. **A**. Representative images of 3D organoids and 2D organoids observed with a bright-field microscope after 8 days and 3 days of culture, respectively. Scale bars = 200 µm. **B**. and **C**. Confocal microscopy images of 3D and 2D organoids stained with antibodies targeting ZO1, Lyz, Lgr5, and VIL1, which mark the apical poles, Paneth cells, stem cells, and enterocytes, respectively. Lgr5 immunostaining of the 2D organoids was performed with an Olympus CKX41, and all the other images were taken with an Operetta CLS (Revvity). The scale bars of the 3D organoid immunostaining with Lyz and Vil represent 200 µm. All the other scale bars represent 100 µm. Nuclei are stained with DRAQ5 (red) for 3D organoids and Hoechst (blue) for 2D organoids. The arrows indicate ZO1 and Lgr5. **D**. Heatmap and hierarchical clustering of the log base e of 1 + FPKM values of transcripts encoding for a selection of biomarkers associated with the different cell types of the intestinal epithelium. If a biomarker corresponds to several isoforms, its number is specified in brackets.

To produce 3D organoids, the jejunum tissues of three stillborn piglets were recovered, and intestinal crypts containing stem cells were isolated from their jejunum tissues and cultivated as described in the Materials and methods section (Figure 1). The crypts evolved from a tubular morphology on the first day after isolation to spheroids after 5 days of culture and finally to 3D organoids after 8 days of culture (Additional file 2A). Regardless of the donor piglet, the 3D organoids reconstituted a highly convoluted intestine-like mini-architecture of the intestine with evaginations and invaginations (Figure 2A), which represent the crypts and villi, respectively. To maintain organoid viability and good growth conditions, the organoids were regularly passaged with an organoid dissociation step followed by reseeding in Matrigel and specific culture medium every week. After 25 passages, the 3D organoids were still able to grow, and we did not observe any morphological differences between the different passages (Additional file 2A).

#### Polarity of 3D and 2D organoids

The polarity of the 3D organoids, which is an apical-in polarity (i.e., the apical pole is localized inside, facing the lumen of the organoid), was determined with a marker of the apical pole, zonula occludens 1 (ZO1), which is localized to tight junctions (Figure 2B) [32]. This apical-in conformation is not compatible with viral infection via the apical pole [25]. Thus, it is essential to make the apical pole accessible to the virus [25]. One experimental solution involves converting 3D organoids into a 2D culture plate. In our study, 3D organoids (passage 25) were dissociated and placed in contact with a Matrigel-coated plate for three days to proliferate and produce a 2D monolayer of cell islands (Figure 2A). The presence of tight junctions was verified in 2D organoids, and ZO1-labelled cells were observed (Figure 2B and Additional file 2B).

#### Cellular diversity of 3D and 2D organoids compared with that of piglet jejunums and crypts

In addition, to assess the cellular diversity expected in the 3D and 2D organoids, immunostaining for cell type biomarkers such as lysozyme (Lyz), villin 1 (VIL1), and leucine-rich repeat-containing G-protein-coupled receptor 5 (Lgr5), which are known to be expressed by Paneth cells, enterocytes, and stem cells, respectively, was performed [16] (Figure 2C). All these biomarkers were detected in 3D organoids, indicating the presence of these different cell types. The results were similar for the three donor piglets. In 2D organoids, VIL1 staining was very abundant, and VIL1 labelling appeared diffuse in the cells, except in the nucleus (Additional file 2C). Lysozyme biomarker staining revealed coarse granules in the cells, preferentially localized inside the 2D organoid islands, in contrast to Lgr5 staining, which was detected mainly at the periphery of the 2D organoid islands (Figure 2C).

In parallel, a comparison of the expression of transcripts encoding specific biomarkers of the different cell types was performed for piglet jejunums, crypts, and 3D and 2D organoids (Additional file 3). At least one biomarker per cell type was expressed, confirming the cellular diversity in the piglet jejunums, crypts, 3D organoids from the 5^th^ to the 25^th^ passages and 2D organoids (Figure 2D). The expression of the transcripts encoding the biomarkers, calculated as log base e of (1 + FPKM) values, started at 0.04 for peptide YY (PYY) (enteroendocrine cells) in 3D organoids after 14 passages and 2D organoids and for sucrase isomaltase (SI) (enterocytes) in 2D organoids. A higher level of expression, at a value of 9.15, was detected for olfactomedin4 (OLFM4) (stem cells) in 3D organoids after 25 passages. The expression of transcripts encoding other biomarkers is presented in the supplemental data (Additional file 2D). The expression of biomarkers, including stem cells (OLFM4, Lgr5, and AXIN2), goblet cells (MUC2), Paneth cells (Lyz), enteroendocrine cells (CHGA), and enterocytes (VIL1 and KRT20), was maintained in the piglet jejunums and 3D organoids at different passages. However, we observed two marker genes whose expression decreased in the organoids compared with that in the jejunums. The first is sucrase isomaltase (SI), whose expression decreased by a factor of 6 (on a log base e scale) between the piglet jejunums and 3D organoids at passage 25. The second is PYY, whose expression continuously decreased during successive 3D organoid passages to a 3-log base e difference in the expression of the PYY (1) transcript after the 25^th^ passage compared with that in the piglet jejunums. The 2D organoids expressed stem cell biomarkers (OLFM4, AXIN2, and Lgr5), enterocyte biomarkers (VIL1 and KRT20), and an enteroendocrine cell marker (CHGA) at the same level as the 3D organoids at passage 25 did. The goblet cell (MUC2) and Paneth cell (Lyz) markers were expressed at a lower level (by log factor of 2) in 2D organoids than in 3D organoids at passage 25.

We compared the expression of the same biomarkers in ST cells, which are commonly used to study TGEV infection. Among the studied biomarkers, only VIL1 and SI were expressed in ST cells (Figure 2D). The expression of these genes was also checked in another published RNA-seq dataset (GEO accession number: GSE136705) of ST cells (Figure 2D and Additional file 3).

Mature enterocytes can transport nutrients from the lumen to the intracellular compartment [33]. This requires the expression of transporters of the solute carrier (SLC) family. The overall expression pattern of SLC family members was equivalent in the different models and the different passages of the organoids (Additional files 4 and 5). The expressed SLC family members are involved in the transport of sugars, fatty acids, steroids and vitamins. In particular, the expression of SLC27A6, a protein involved in lipid transport, was similar in 3D (after 14 passages) and 2D organoids and piglet jejunums. In contrast, ST cells did not express SLC17A7, a glutamate transporter (Additional files 4 and 5).

### Variability in transcript expression among biological replicates of in vivo, in vitro, and organoid models

During in vivo experiments, significant variability is often observed between individuals, and this variability can sometimes compromise the robustness of downstream statistical analyses, particularly when the number of replicates is low, which is often the case for experiments involving large animals (such as pigs).

**Figure 3:**
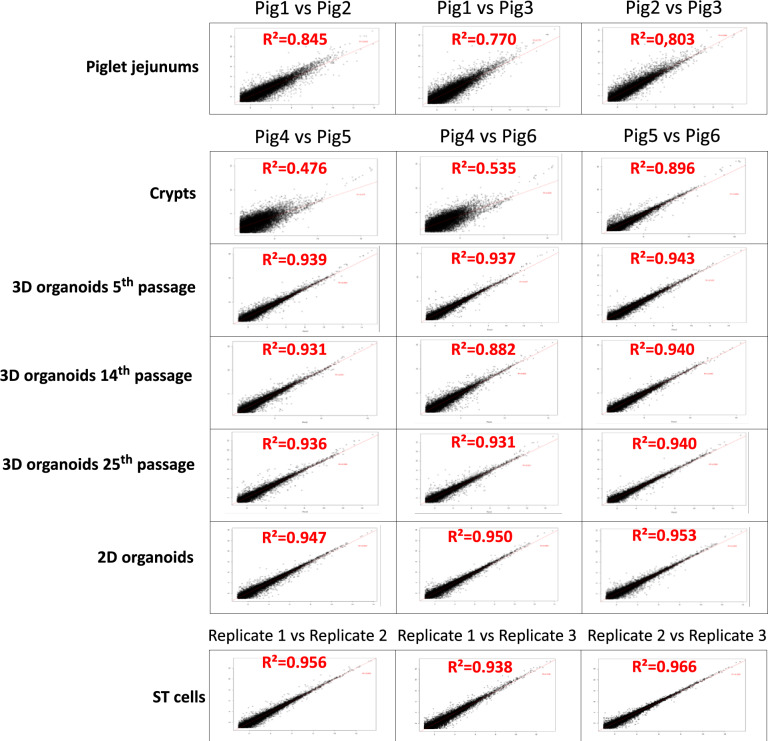
**Analysis of the expression variability among biological replicates**. After RNA sequencing, the reads were cleaned and mapped onto the pig transcriptome. The FPKM values were subsequently calculated, and only the transcripts with a log_2_-scale FPKM greater than 1 in at least one sample were retained. If log_2_(FPKM) < 1, a log_2_(FPKM) = 0 is displayed. Scatter plots were constructed to display the expression profiles of the replicate pairs. Determination coefficient values were then calculated on the basis of the log_2_-scale FPKM of the biological replicates to quantify expression variability. Coefficients of 1 and -1 represent perfectly correlated and anti-correlated profiles, respectively.

To determine the extent of this variability among biological replicates of each experimental model (piglet jejunums, crypts, 3D and 2D organoids, and ST cells), we calculated the determination coefficient (R^2^) value between the respective transcripts of each biological replicate expression profile. To do so, the transcript expression levels of all the biological replicates were collected as FPKM values (see Materials and methods) (Figure 3 and Additional file 6). The determination coefficient (R^2^) was calculated among the three SPF 5-week-old piglet jejunums, and the corresponding R^2^ values varied between 0.770 and 0.845. For the crypt models, we observed that the crypts from pig 4 had a different transcript expression profile compared to those from pigs 5 and 6 (R^2^ values of 0.476 and 0.535 in the comparison with pig 5 and pig 6, respectively). Pigs 5 and 6 had an R^2^ value of 0.896. Even if variations existed among biological replicates in crypts used to derive organoids, a lower level of interreplicate variability was observed after only five passages for 3D organoids (determination coefficient of 0.94). The homogeneity of transcript expression was maintained after 14 passages, with R^2^ values ranging from 0.882 to 0.940. Moreover, after the long-term culture of 25 passages, this lower variability (R^2^ = 0.940) was maintained. In addition, the 2D organoids derived from these 3D organoids were associated with low interreplicate variations in transcript expression (R^2^ = 0.950). Ultimately, and as expected for the cellular model, the R^2^ for the ST cells was also high (values ranged from 0.956 to 0.966 at the same passage). R^2^ values were also calculated for gene expression to validate our observations (Additional file 7). Although the use of gene expression resulted in fewer differences among replicates, these results were consistent with those obtained with transcript expression (determination coefficient of 0.924 between the two sets of R^2^).

### Comparison of transcript expression profiles among in vivo, in vitro and organoid models without infection

On the basis of the analysis of transcript expression among biological replicates at different passages of the 3D organoids, we demonstrated long-term expansion with low transcript expression variability. Furthermore, the expression of cellular biomarkers did not vary significantly across passages, suggesting the maintenance of cell type diversity, even at the 25^th^ passage. Therefore, we focused on the analysis of 3D organoids at the 25^th^ passage and 2D organoids produced from this passage. To analyse the relevance of the 3D and 2D organoids as experimental models, their transcript expression as determined by RNA-seq was compared with the profiles of piglet jejunums, crypts, and ST cells. For all the models and for each transcript, the average of the log_2_(FPKM) values across all replicates was calculated and used in the downstream analyses.

**Figure 4:**
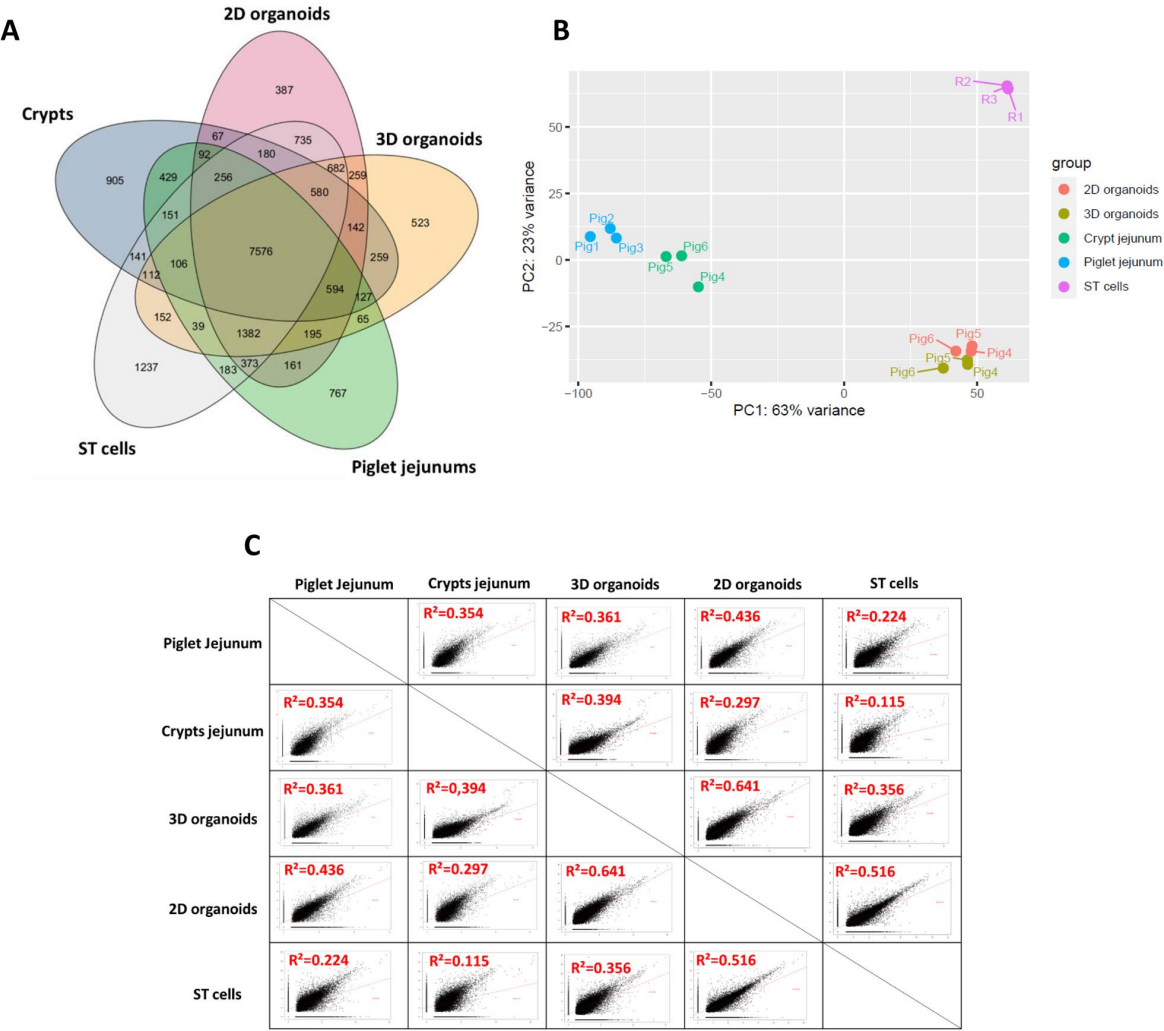
**Comparisons of transcript expression among piglet jejunums, crypts, 3D and 2D organoids and ST cells**. The average log_2_(FPKM) value was calculated, and for each experimental model, log_2_(FPKM) values were defined as log_2_(FPKM) > 1 and padj < 0.05. **A**. Venn diagram representing log_2_(FPKM) > 1 and padj < 0.05 for each experimental model. **B**. Principal component analysis based on the read counts of the five experimental models. **C**. Determination coefficient (R^2^) values calculated between the log_2_(FPKM) values of the experimental models.

To visualize the comparison of transcript expression of the different experimental models, we used a Venn diagram that shows the number of transcripts expressed specifically in each model or shared between models (Figure 4A). An average of 12 904 transcripts were expressed in piglet jejunums, crypts, 3D organoids, 2D organoids, and ST cells. The crypts expressed the lowest number of transcripts (11 717 transcripts), and the ST cells expressed the highest number (13 885 transcripts).

In addition, PCA revealed a specific transcript expression pattern for each model (Figure 4B). The first two components captured 63% and 23% of the total variance, respectively, representing the vast majority (86%) of the variations detected between the different samples. In this representation, the biological replicates of each model clustered together, highlighting the homogeneity of the generated data. In addition, the 3D and 2D organoid samples also clustered in very close proximity. Porcine jejunums, crypts and ST cells were distant from the 3D and 2D organoids.

Finally, R^2^ values were computed to compare transcript expression levels between the models (Figure 4C). The highest R^2^ value was observed between 3 and 2D organoids (R^2^ = 0.641), and the lowest value was observed between ST cells and jejunum crypts (R^2^ = 0.115). Piglet jejunums had R^2^ values of 0.361 and 0.436 for 3D and 2D organoids, respectively. The R^2^ value for the ST cells and 2D organoids was 0.516. The 3D organoids from the 5^th^ and 14^th^ passages, which had a R^2^ value of 0.876 between them, had strong R^2^ values of 0.791 and 0.878, respectively, with the 25^th^ passage (Additional file 8).

### Analysis of gene function in in vivo, in vitro, and organoid models without infection

#### Functions of expressed transcripts shared in all experimental models

As observed previously, the 3D and 2D organoid models recapitulated the cellular diversity of the intestinal epithelium. To better characterize the 3D and 2D organoid models, the expression of transcripts specific to each model or shared between them was analysed. On the basis of the Venn diagram (Figure 4A), 7576 transcripts were expressed in all five models, corresponding to 40.1% of the 18 907 transcripts identified in at least one model. As expected, these broadly expressed transcripts encoded genes enriched in essential housekeeping functions for cell viability, such as the regulation of mRNA processing (DDX17, RBFOX2, DDX23, and HNRNPA1), ribonucleoprotein complex biogenesis (PWP1, TARBP2, PA2G4, and PTGES3), and ribosome biogenesis (UTP20, RIOK1, PAK1IP1, and RPL10A) (Additional file 9, Additional file 10A).

#### Functions of expressed transcripts shared by the piglet jejunums, crypts, and 3D and 2D organoids

**Figure 5:**
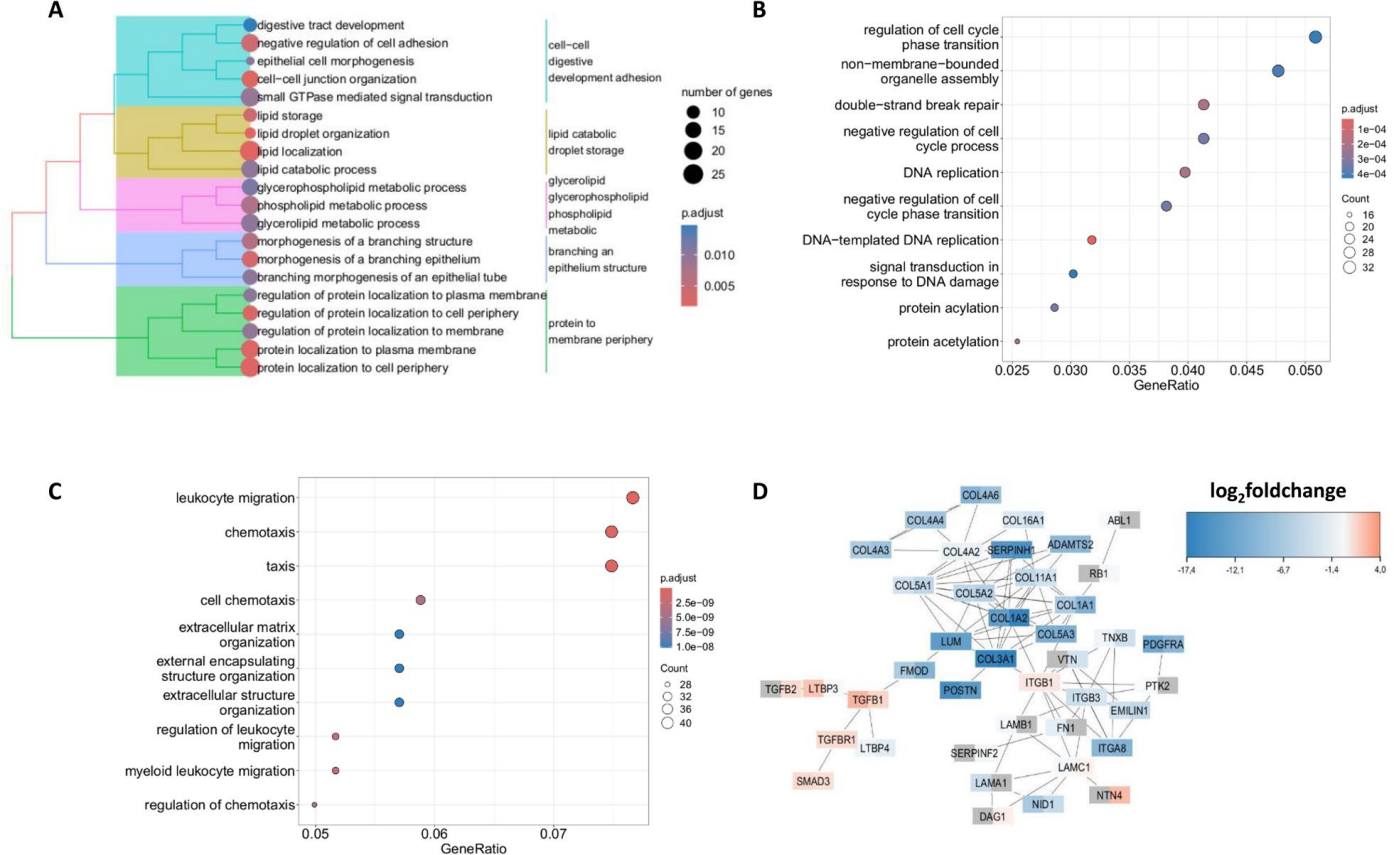
**Functional analysis of gene expression for the different experimental models**. Functional enrichment was performed with the molecular function tree of Gene Ontology and by considering only the transcripts whose log 2-scale FPKM was greater than 1. Functional terms associated with an adjusted *P* value greater than 0.05 were discarded. **A**. Functional enrichment of the genes expressed in crypts, piglet jejunums, and 3D and 2D organoids. Similar Gene Ontology terms were grouped into more generic functional categories (corresponding to different colours). **B**. Functional enrichment of genes expressed in 2D organoids and ST cells. **C**. Functional enrichment of the genes expressed only in piglet jejunums. **D**. Differential gene expression analyses between 3D/2D organoids and piglet jejunums were performed. Genes associated with extracellular matrix organization with at least one adjusted *P* value less than 0.05 and at least one log_2_-fold change greater than 2 (or less than -2) were included in the network. Network view representing the genes involved in extracellular matrix organization. For each network node, corresponding to a gene, the left side of the node represents the log_2_-fold change in 3D organoids, whereas the right side represents the log_2_-fold change in 2D organoids. The grey colour represents inconclusive data (e.g., *P* > 0.05).

Piglet jejunums, crypts, and 3D and 2D organoids also shared the expression of 594 genes, corresponding to 3.14% of the 18 907 total transcripts. A Gene Ontology analysis revealed enrichment of genes involved in epithelial development (GO:0045216, GO:1990778, and GO:0048754), localization of protein to the membrane periphery (GO:0072659, GO:1904375, GO:1903076, and GO:1904377, and GO:1903078) and lipid metabolism (GO:0006650, GO:0016042, GO:0046486, GO:0034389, GO:0010876, and GO:0019915) (Figure 5A and Additional file 9). By focusing on the molecular function subtree, more precise functions related to lipid metabolism, such as phospholipid binding (GO:0005543), phosphatidylinositol binding (GO:0035091), and nucleoside metabolism (GO:0060589 and GO:0005085), were identified (Additional files 9 and 10B). A similar analysis was carried out with the human annotations. This revealed Gene Ontology terms equivalent to those found with the mouse annotations (Additional file 11A).

Piglet jejunums, crypts, and 3D and 2D organoids also shared the expression of genes implicated in the morphogenesis of a branching epithelial structure (Figure 5A). To identify these genes and to show the similarities and differences between our models, the genes implicated in this biological process were recovered and analysed. The log_2_-fold changes in the crypts and 3D and 2D organoids compared with those in the piglet jejunums were calculated. Among these genes involved in intestinal cell renewal and stem cell maintenance, genes that were upregulated in the crypts and in 3D and 2D organoids compared with piglet jejunums, such as Wingless Type 7 B (WNT7B) and Sry Box Transcription factor 9 (SOX9), whose expression levels were ten and three times greater, respectively, in 3D and 2D organoids than in piglet jejunums, were observed. Other members of the WNT and SOX pathways, such as SOX11, WNT6, and WNT7A, were upregulated only in 3D and 2D organoids, in contrast to crypts, in which the genes were not deregulated compared to piglet jejunums (Additional file 12).

Among the 566 genes involved in morphogenesis, 174 and 162 genes were downregulated in 3D and 2D organoids, respectively, and 34 were downregulated in crypts compared with piglet jejunums. All three models exhibited the downregulation of genes encoding ACTA2, an isoform of actin that plays a role in the contraction of intestinal smooth muscle, and GREM1, an antagonist of BMP expression [34, 35]. Downregulated genes such as BMP5, BMP3, and PDGFRA, which are involved in the differentiation of villus cells, and some genes involved in matrix organization, such as collagen 3A1 (COL3A1), serpin family H member 1 (SERPINH1) and multimerin2 (MMRN2), were observed in 3D and 2D organoids in comparison with piglet jejunums (these genes not found in crypts). The downregulation of genes involved in the BMP pathway was due to the presence of noggin in the organoid culture medium [36].

#### Functions of expressed transcripts shared by 3D and 2D organoids, ST cells and piglet jejunums

3D and 2D organoids shared the expression of 195 transcripts (corresponding to 1.07% of the 18 907 total transcripts) with the piglet jejunums and 682 transcripts with ST cells (corresponding to 3.60% of the 18 907 total transcripts). Therefore, to determine the common functions between these experimental models, a Gene Ontology analysis was performed. Among 195 transcripts that were expressed only in piglet jejunums, 3D, and 2D organoids (therefore absent in crypts and ST cells), 20 genes were involved in ERB2–ERB3 signalling (GO:0038133 and GO:0038129), wound healing (GO:0042060), positive regulation of dendrites (GO:0050775), and cell morphogenesis (GO:0010770) (Additional file 9 and Additional file 10C). ST cells expressed 682 transcripts in common with those in 3D and 2D organoids, which were enriched in RNA localization and transport (GO:006403, GO:0051236, GO:0050658 and GO:0051028), stress-activated protein kinase signalling (GO:0031098 and GO:0051403), ncRNA transcription (GO:0098781), and miRNA metabolic process (GO:0010586 (Additional file 10D). 2D organoids and ST cells shared the expression of 735 transcripts, and Gene Ontology analysis revealed their enrichment in the regulation of cell cycle phase transition (GO:1,901,988, GO:0010948, GO:1,901,987, and GO:0044839), DNA replication (GO:0006261 and GO:0006260), double-strand break repair (GO:0006302 and GO:0042770) and protein acylation/acetylation (GO:0006473 and GO:0043543). (Figure 5B and Additional file 9). A similar analysis was carried out with the human annotations. The Gene Ontology terms identified were equivalent to those identified with the mouse annotations (Additional file 11B). However, the genes shared by the 3D organoids and ST cells showed no enrichment according to the Gene Ontology analysis.

#### Functions of transcripts expressed only in the piglet jejunums

As shown above, the genes whose expression was present in different models either corresponded to broad functions (housekeeping genes) or to functions specific to a tissue (e.g., epithelial development), but each model also displayed specific transcribed genes. Indeed, the piglet jejunum was the only model associated with an enrichment of genes associated with leukocyte migration (ITGB7, ADTRP, GSPM3, PLA2G7, and IL16) and extracellular matrix organization (genes encoding different types of collagen (COL), laminin (LAM), or integrin (ITG)) (Figure 5C and Additional file 9). A similar analysis was carried out with the human annotations, and the corresponding results were equivalent to those of their mouse counterparts (Additional file 11C). To better understand the differences in the expression of genes encoding the proteins constituting the extracellular matrix organization between piglet jejunums and organoids, the associated genes were recovered, and a gene network was constructed. The genes encoding collagen isoforms (COL1A1/2, COL4A2/3/4/6, COL5A1/2/3, COL3A1, and COL11A1) were downregulated in both 3D and 2D organoids (Figure 5D). Other constituents of the extracellular matrix, such as elastin (EMILIN1), laminin (LAMA1, LAMB1, and LAMC1), fibronectin (FN1), fibromodulin (FMOD), lumican (LUM), and periostin (POSTN), were downregulated. Integrin subunit beta 3 (ITGB3), a member of the integrin family that plays a role in cell adhesion by binding to collagen, fibronectin, and laminin, was also downregulated. Despite the repression of genes encoding extracellular matrix components, the expression of genes involved in the TGF beta 1 signalling pathway, including LTBP3 and SMAD3, which are linked to the extracellular matrix, was upregulated in 3D organoids (Figure 5D).

#### Functions of transcripts expressed only in 3D organoids and ST cells

The piglet jejunums, 3D and 2D organoids and ST cells presented specific transcript expression profiles (Figure 4A). The 3D organoids expressed 523 transcripts (2.76% of the 18 907 total transcripts), which were enriched in functions related to response to nutrient levels (GO:0031667 and GO:0031669), response to extracellular stimulus (GO:0009991 and GO:0031668), Golgi vesicle transport (GO:0048193 and GO:0006892), cellular response to starvation (GO:0009267), response to topologically incorrect proteins (GO:0035966 and GO:0035967), and developmental growth involved in morphogenesis (GO:0060560) (Additional file 9 and Additional file 10E). The ST cells expressed 1237 specific transcripts (6.54% of the 18 907 total transcripts), with a Gene Ontology enrichment in cilium organization and assembly (GO:0060271 and GO:0044782), regulation of synapse structure and organization (GO:0050807 and GO:0050803), microtubule-based transport (GO:0099111), and protein localization to the cilium (GO:0061512) (Additional file 9 and Additional file 10F). The 387 transcripts (2.05% of the 18 907 total transcripts) expressed only in 2D organoids did not provide any Gene Ontology enrichments higher than the *p* value of 0.05.

### Characterization of the functional infection of 2D organoids by TGEV

To evaluate the suitability of organoids for the study of host–virus interactions, infections with transmissible gastroenteritis virus (TGEV) were tested. Infections were performed on 2D organoids to allow adhesion and entrance of the virus via the accessible apical pole.

To infect a cell, TGEV needs to bind a viral receptor, porcine aminopeptidase N (pAPN), with the help of cofactors such as EGFR and sialic acid [24, 37]. To verify that the 2D organoids produced from 3D organoids at passage 25 had an accessible pAPN, immunostaining was performed (Additional file 13A). Immunostaining revealed pAPN in 2D organoids as coarse granules in the cytoplasm and membrane of the cells, confirming the expression of the main viral receptor protein. In addition, the expression of genes encoding these receptors in 2D organoids was determined by RNA-seq analysis, which revealed gene expression reaching 4 log_2_ (Additional file 13B).

**Figure 6:**
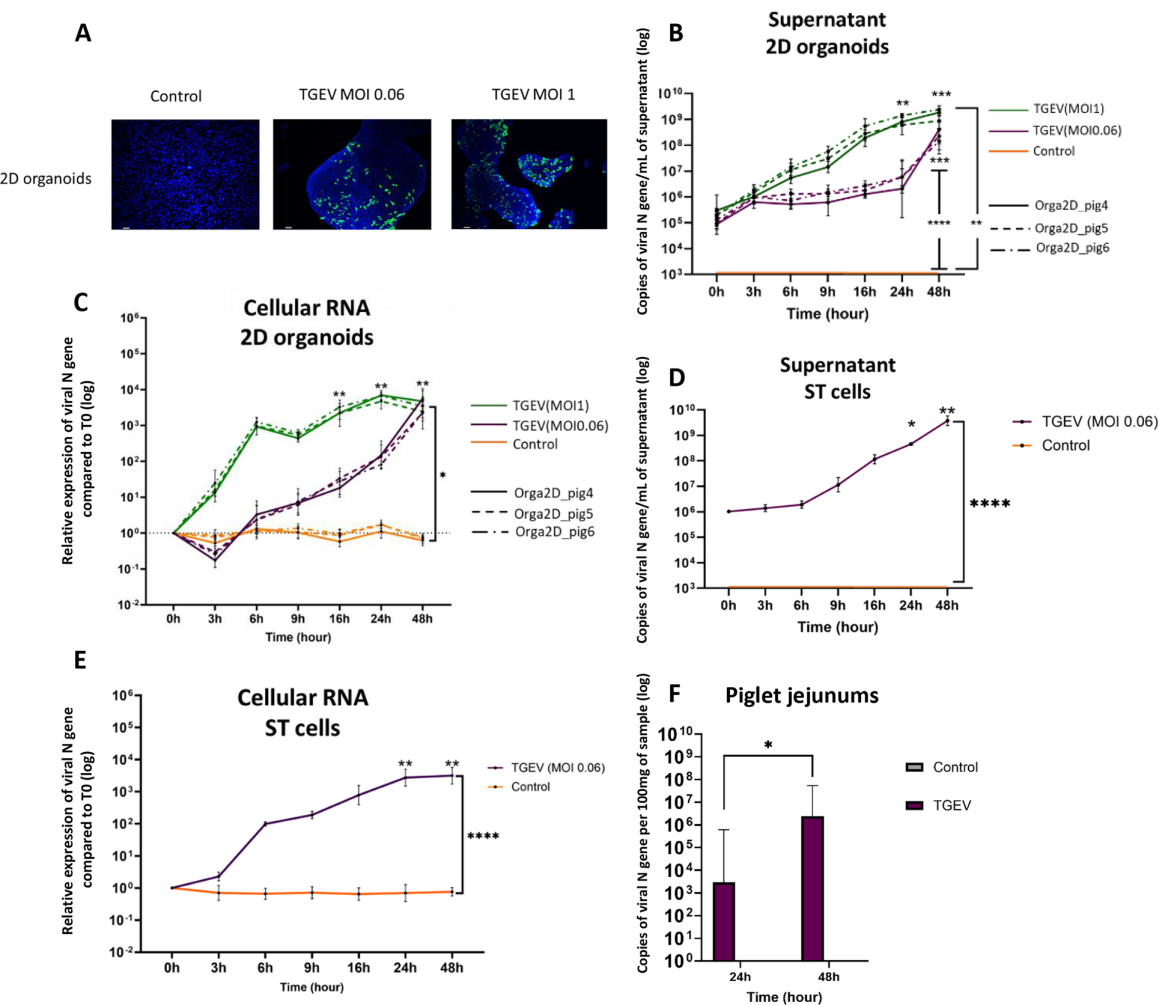
**Infection of the experimental models**. The kinetics of infection were determined for the ST cells (*n* = 3) and 2D organoids (*n* = 4 for each donor piglet) infected at an MOI of 0.06. In parallel, an MOI of 1 was also tested in 2D organoids. Piglet jejunums were infected with TGEV at 10^8^ copies of the viral N gene. **A**. At 24 hpi, 2D organoids were fixed, permeabilized and stained for the TGEV N protein (green) and nuclei (blue). Images were obtained with an Olympus CKX41 fluorescence microscope. Scale bar = 100 µm. **B** and **D**. Supernatants of ST cells (**B**) and 2D organoids (**D**) were harvested at different time points, RNA was extracted, and RT‒qPCR targeting the viral N gene was performed. The detection limit of RT‒qPCR was 10^3^ copies of the viral N gene/mL **C**, **E** and **F**. Cellular RNA was collected at different time points and analysed by RT‒qPCR. The relative expression of the viral N gene at the first time point was calculated and normalized to that of GAPDH and HPRT1 for the 2D organoids (**C**) and ST cells (D). The number of copies of the viral N gene per sample of piglet jejunum was calculated (F). *, *P* < 0.05; **, *P* < 0.01; ***, *P* < 0.001; ****,* P* < 0.0001.

To visualize viral infection, immunostaining for viral nucleoprotein (N) was performed at 24 hpi (Figure 6A) in infections at an MOI of 0.06 or 1. The presence of the viral N protein was detected in some of the cells on the entire surface of the 2D organoid islands. No fluorescence was detected in the control samples.

The supernatant of the infected organoids was harvested, and RNA was extracted to assess the genomic and subgenomic viral N gene by nondiscriminating RT‒qPCR (Figure 6B). The dynamics of viral production were similar between all the donor piglets for infection at each MOI (1 and 0.06). An increase in viral N gene copy number was observed after infection at an MOI of 0.06 from 0 to 48 hpi, with a maximum viral production of 3 log_10_. With infection at an MOI of 1, an increase in the viral N gene was also observed, with a maximum viral production of 4 log_10_ from 0 to 48 hpi.

In parallel, cellular RNA was extracted, and the viral N gene was assessed by RT‒qPCR (Figure 6C). For infection at an MOI of 0.06, a constant increase in the viral N gene of 3 log_10_ was observed between 0 and 48 h. At an MOI of 1, a 3 log_10_ increase compared with that at 0 h was obtained at 6 hpi. After 24 hpi, a relative expression of 4 log_10_ compared with that at 0 h was observed at an MOI of 1. At 48 hpi, at an MOI of 1, close to 80% of the cells in the 2D organoids had died. We also detected similar infection dynamics among the three donor piglets at MOIs of 0.06 and 1. Interestingly, the same capacity for replication was achieved at 48 hpi, with a relative expression of the viral N gene of 3.6 log_10_ between the two MOIs (Figure 6C).

### Comparison of TGEV infection in 2D organoids with that in ST cells and piglet jejunums

For comparison with 2D organoids, ST cells and eight piglets were infected.

In the ST cell supernatant, a significant increase in the viral N gene was observed between 0 and 48 h, with a viral yield of 3 log_10_ (Figure 6D).

We analysed the cellular RNA of ST cells and piglet jejunums at all time points (Figures 6E and F). With respect to the 2D organoids, the relative expression of the viral N gene was determined by comparison at each time point to that at 0 hpi and normalized to GAPDH expression for the ST cells. A progressive and significant increase in the viral N gene was observed after the initial infection, reaching a maximum of 3 log_10_ at 24 hpi (Figure 6E). In jejunum tissues, we observed an increase of 3 log_10_ of the viral N gene between 24 and 48 hpi (Figure 6F). The viral N gene was also detected in the duodenum of all piglets at 48 hpi, whereas only two piglets were positive in the ileum. A small number of copies of the viral N gene (close to the detection limit of RT‒qPCR of 10^3^ copies of viral N gene/mL) was detected in the faeces and serum of infected pigs (data not shown). The virus was also detected in the respiratory complex, particularly in the bronchial tubes (an average of 10^7^ copies of the viral N gene at 24 hpi and 48 hpi) and tracheobronchial lymph nodes (an average of 10^5^ and 10^7^ copies of the viral N gene at 24 hpi and 48 hpi, respectively) (Additional file 14).

### Comparison of the transcript expression levels among the infected experimental models

We previously reported that the dynamics of infection were similar between donor pigs at each MOI. We analysed whether gene expression was also homogeneous at two time points during infection. To do so, we performed RNA-seq analysis at the 9 hpi time point for ST cells and 2D organoids to detect cellular responses during the first viral cycle. In addition, we maintained the 24 hpi time point, which corresponds to multiple viral cycles, to compare all the experimental models fairly.

**Figure 7:**
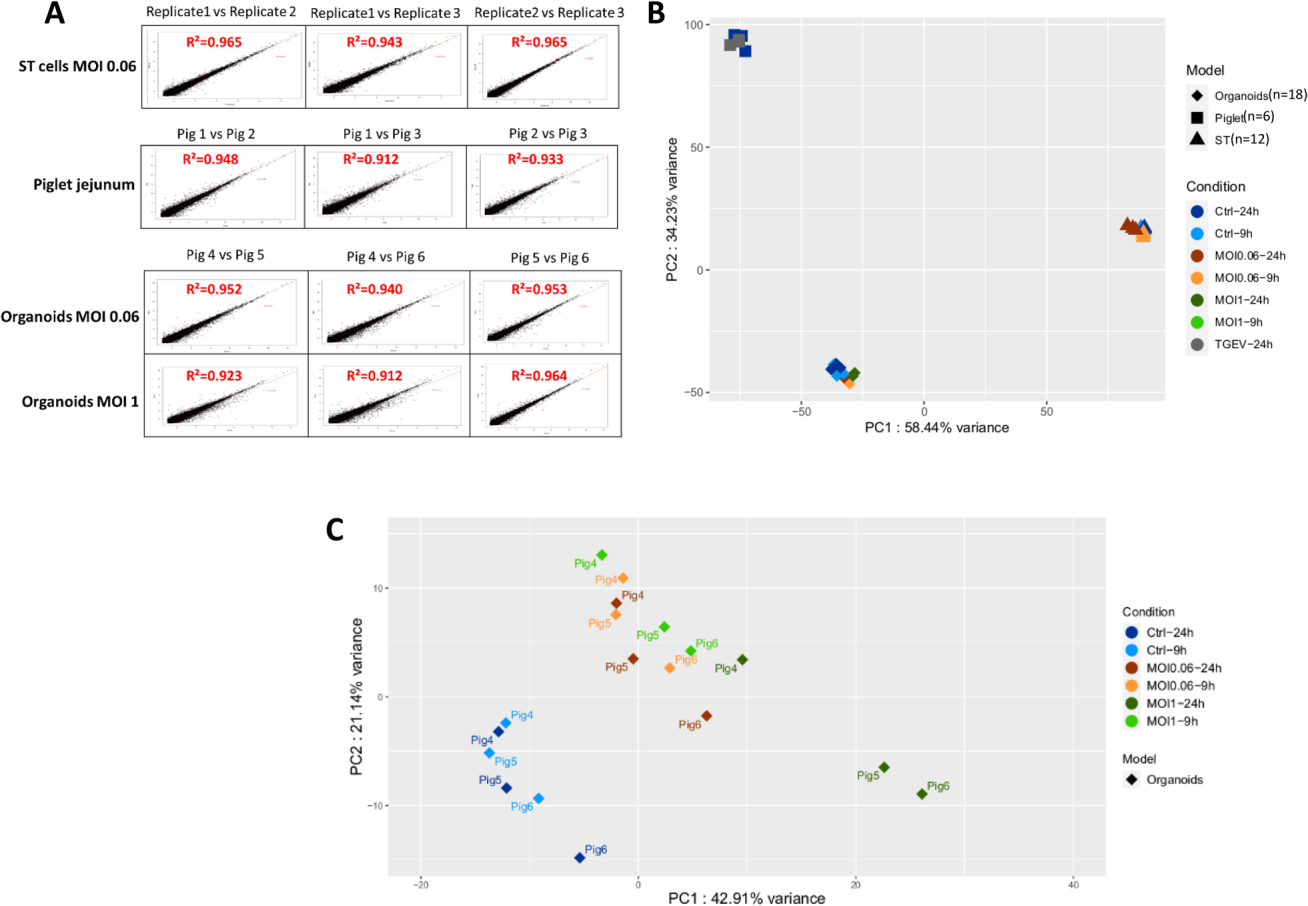
**Comparison of the expression profiles of the infected experimental models.** For each experimental model, the average across replicates was calculated, keeping only the transcripts with a log_2_-scale FPKM greater than 1. **A**. Scatter plots and Pearson correlation coefficients calculated between the log_2_-scale FPKM values of infected experimental model pairs at 24 hpi. **B**. Principal component analysis based on the normalized read counts of the control and infected experimental models. All the models were analysed at the 24 hpi time point. In addition, an earlier time point, corresponding to 9 hpi, was also included for the ST cells and 2D organoids. **C**. Principal component analysis based on the read counts of the infected and control 2D organoids.

We calculated the variability in gene expression between infected biological replicates at 24 hpi with determination coefficient (R^2^) values (Figure 7A). Small variabilities were observed for the three infected models, with R^2^ values ranging from 0.912 to 0.965. Indeed, the R^2^ values were close to and slightly higher than the noninfected models with an average R^2^ value of 0.965 for infected ST cells (0.953 for noninfected ST cells), 0.931 for infected piglet jejunums (0.806 for noninfected piglet jejunums), and 0.948 and 0.933 for 2D organoids infected at MOIs of 0.06 and 1, respectively (0.95 for the noninfected 2D organoids).

To visualize the effect of infection on gene expression, we performed a PCA with the read counts of both infected and noninfected samples (Figure 7B). The first two components captured 58.44% and 34.23% of the total variance, respectively. In this representation, the samples, whether infected or not, were still clustered according to the experimental model of origin. However, when PCAs were carried out on separate experimental models, the infected and control samples did not cluster together (Figure 7C and Additional file 15). The ST cell and 2D organoid samples began to diverge from their respective control samples after 9 hpi. However, the greatest difference was observed at 24 hpi for all the models. This analysis thus suggests that there were differences in gene expression not only between the different models but also for each model between the control and infected samples (Figure 7C and Additional file 15).

### Differential gene expression profiles induced in the three infected experimental models

To understand which genes were differentially expressed in each model, log_2_-fold changes were calculated by comparing the infected and control samples. Genes associated with an adjusted *P* value lower than 0.05 and a log_2_-fold change greater than 1 or less than −1 were considered upregulated or downregulated, respectively.

**Figure 8:**
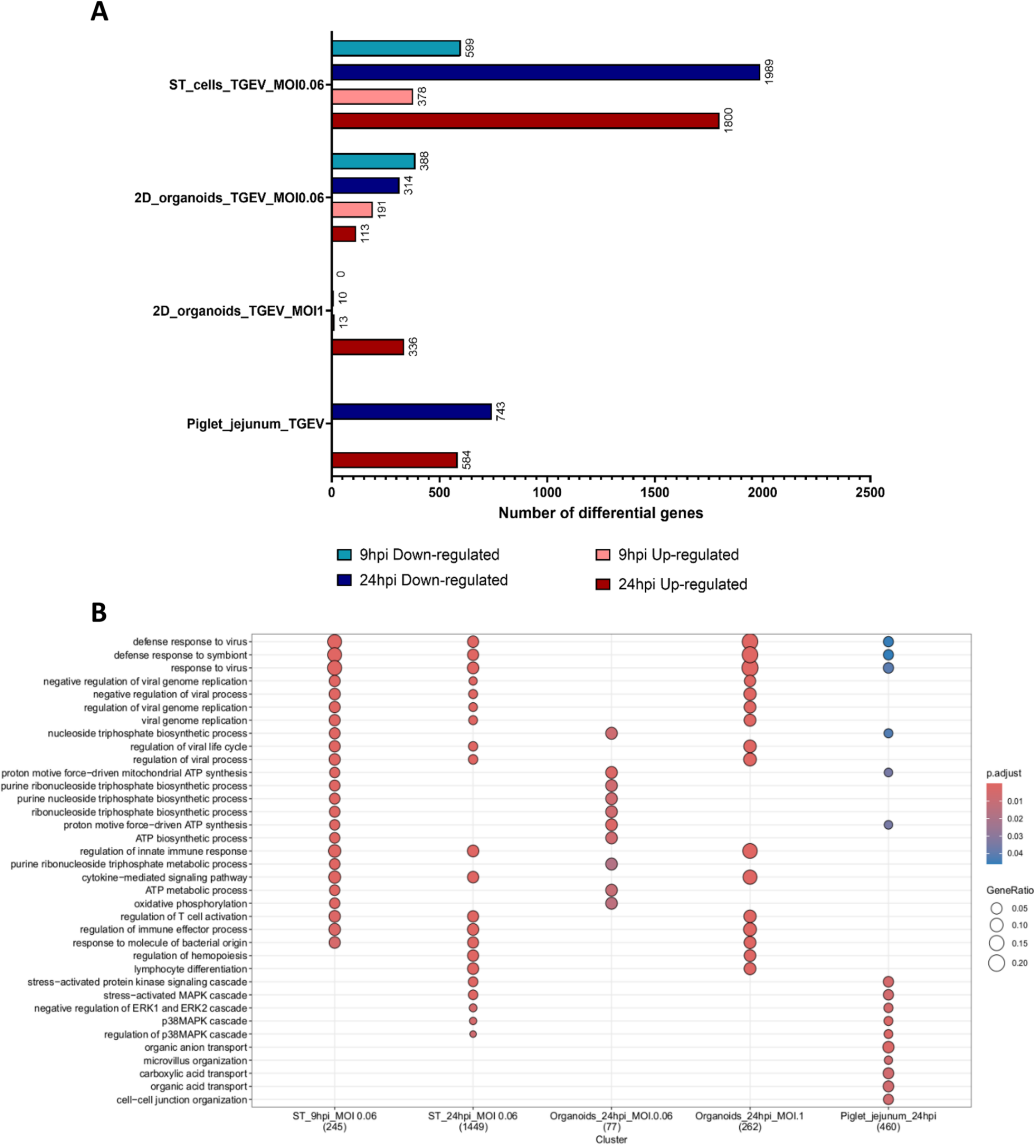
**Differential gene expression analysis between control and infected samples**.** A**. Number of differentially expressed genes detected for the different time points and experimental models. A gene was considered to be differentially expressed when its adjusted *P* value was less than 0.05 and its log_2_-fold change was either greater than 1 (upregulated) or less than -1 (downregulated). **B**. Functional enrichment of the upregulated genes in each infected experimental model. The 9 hpi time point for the infected 2D organoids was not represented, as no enrichment was detected.

The number of differentially expressed genes was calculated at 9 hpi for the ST cells and 2D organoids and at 24 hpi for all the experimental models (Figure 8A). At 9 hpi, the infected ST cells downregulated 599 genes compared with 388 genes in the 2D organoids infected at an MOI of 0.06. No downregulated genes were detected in 2D organoids infected at an MOI of 1 at 9 hpi according to our criteria. In addition, at 9 hpi, 378, 191, and 13 genes were upregulated in ST cells, 2D organoids infected at an MOI of 0.06, and 2D organoids infected at an MOI of 1, respectively. At 24 hpi, 1989 genes were downregulated in infected ST cells compared with 743 in infected piglet jejunums. With respect to the organoids, 314 and 10 genes were downregulated at MOIs of 0.06 and 1, respectively. Ultimately, at 24 hpi, 1800 genes were upregulated in infected ST cells, which was three times more than that in piglet jejunums, in which 584 genes were upregulated. With respect to the organoids, 113 and 336 genes were upregulated at MOIs of 0.06 and 1, respectively.

### Gene ontology analysis of the deregulated genes of the three infected experimental models

We then performed a functional enrichment analysis with Gene Ontology and, more precisely, the biological process subtree. We first analysed the upregulated genes at both time points (9 and 24 hpi) and for all infected experimental models (Figure 8B and Additional file 16). Unfortunately, no enrichment was detected at the 9 hpi time point for 2D organoids, regardless of the MOI. Interestingly, Gene Ontology terms corresponding to viral defence response (GO:0051607, GO:0140546, and GO:0009615) were identified for ST cells (9 hpi and 24 hpi), 2D organoids (MOI of 1, 24 hpi), and piglet jejunums (24 hpi). Moreover, overexpression of the regulation of viral genome replication (GO:1,903,901, GO:0048525, GO:0045071, and GO:0019079) was detected in infected ST cells (at both time points) and 2D organoids (MOI of 1, 24 hpi). We also observed that infected piglet jejunums, organoids (MOI of 0.06, 24 hpi), and ST cells (9 hpi) overexpressed genes involved in nucleoside triphosphate biosynthetic processes (GO:0009142, GO:0009206, GO:0009145, GO:0009201, GO:0009142, GO:0046034, GO:0009205, GO:0009199, GO:0009144, GO:0009141, and GO:0009152) upon infection. Infected ST cells and piglet jejunums at 24 hpi were enriched in the mitogen-activated protein kinase (MAPK) signalling pathway (GO:0051403, GO:0038066, and GO:1,900,744). Infected piglet jejunums (24 hpi) upregulated genes related to microvillus organization (GO:0032528), organic acid transport (GO:0015711, GO:0015849, and GO:0046942), and cell–cell junction organization (GO:0045216). A similar analysis was carried out with the human annotations, and the human and mouse results were equivalent (Additional file 17).

We then performed the same analysis for the downregulated genes. No enrichment was detected in the 2D organoids, regardless of the MOI. However, genes involved in extracellular structure organization (GO:0030198 and GO:0043062) were downregulated in piglet jejunums and ST cells (24 hpi). In addition, ST cells exhibited an enrichment of gene involved in cilium organization (GO:0060271). Finally, genes that participate in DNA replication were downregulated in infected piglet jejunums (GO:0006260) (Additional file 16 and Additional file 18).

### Analysis of the effects of TGEV infection on the intestinal epithelium barrier and the NOTCH pathway

**Figure 9:**
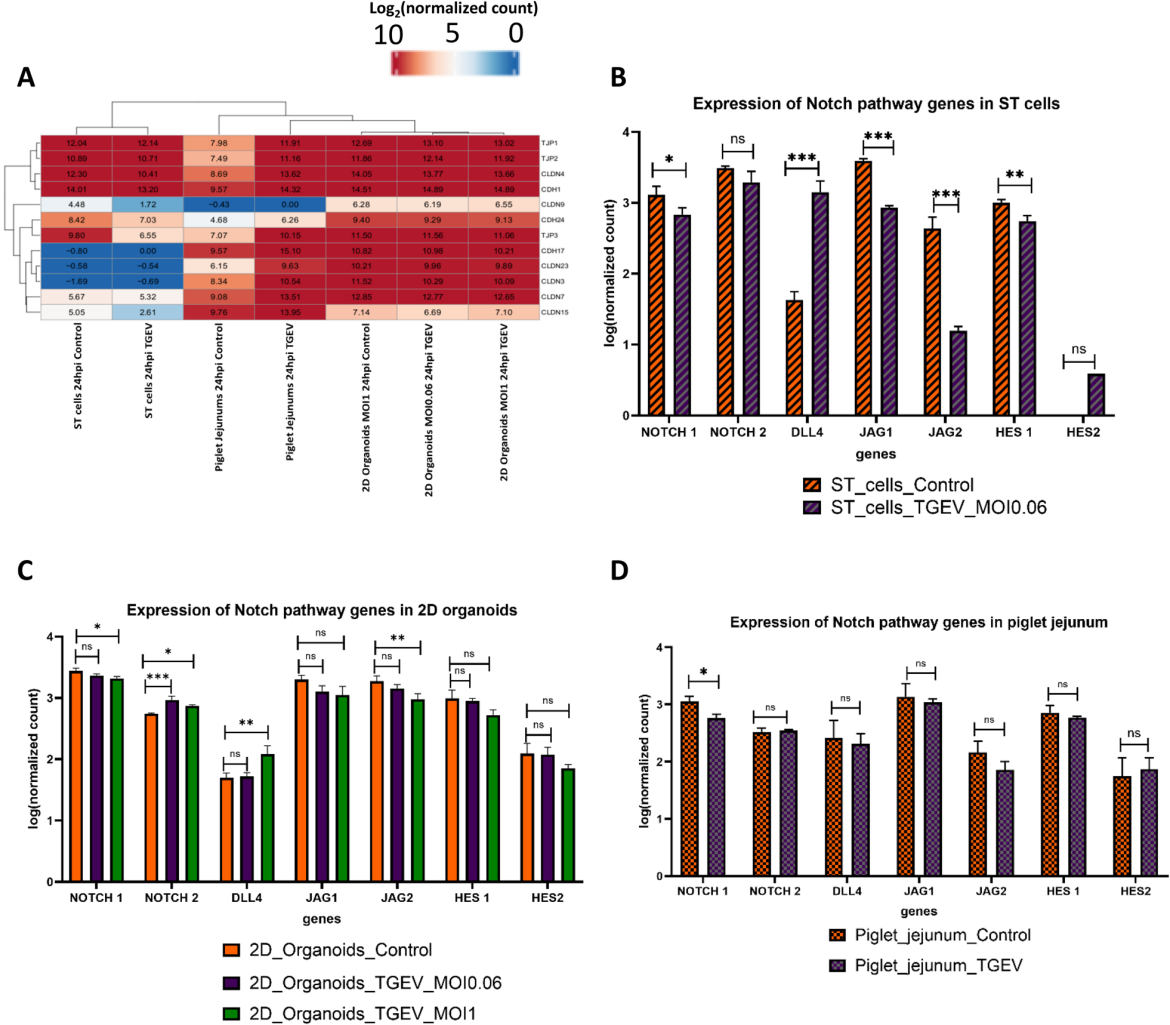
**Expression of genes involved in epithelial barrier function and the NOTCH pathway in control and infected models**. **A**. Analysis of gene expression between control and infected experimental models for genes involved in intestinal epithelium barrier function. **B**–**D** Expression of genes involved in the NOTCH pathway. The average of DESeq2-normalized counts transformed into logs was calculated. Expression of NOTCH pathway genes in ST cells (**B**), 2D organoids (**C**) and piglet jejunums (**D**) in infected and control samples.

To analyse the capacity of 2D organoids to activate the barrier epithelium, the expression of functionally implicated genes was analysed in the control and 24 hpi groups (Figure 9A). Under control conditions, 2D organoids expressed genes encoding tight junction proteins, such as claudins and cadherins. In fact, strong expression of claudin 4 (CLDN4) and cadherin 1 (CDH1) was detected. A marked difference between 2D organoids and piglet jejunums was the expression of claudin 9 (CLDN9), with a difference of 6 log_2_ in favour of 2D organoids. The sequencing results indicated that the ST cells did not express claudin 3 (CLDN3), cadherin 23 (CLDN23), or cadherin 17 (CDH17), whereas the piglet jejunums and 2D organoids did. TGEV infection induced a decrease in claudin 3 (CLDN3) expression in piglet jejunums and 2D organoids.

Cellular diversity of the intestinal epithelium is obtained by the proliferation and differentiation of stem cells through the NOTCH pathway, which is one signalling pathway that controls these processes, notably through the expression of NOTCH1/2, Delta-4 (DLL4), Jagged-1 (JAG1), Jagged-2 (JAG2), and HES1/2 [38]. All these genes were present in control samples of ST cells, 2D organoids, and piglet jejunums. Upon infection of ST cells and organoids (MOI of 1), the expression of NOTCH1 and JAG2 significantly decreased, in contrast to that of DLL4, whose expression increased. In the jejunums of infected piglets, only the decrease in NOTCH1 expression was statistically significant (Figures 9B–D).

### Analysis of gene expression implicated in exocytosis/endocytosis

Viral infection and proliferation are linked to host cellular mechanisms; in particular, viral entry into cells, mediated by endocytosis, and viral exit, regulated by exocytosis [39]. TGEV has an enteric tropism, and analysing the expression of genes that influence viral entry and exit is relevant.

We noticed that some of these genes were specifically expressed in the intestinal epithelium and were thus absent in ST cells, regardless of their infection status. With respect to endocytosis, DLL1, TLR4, and PYCARD were expressed only in piglet jejunums and 2D organoids (Additional file 19A). With respect to exocytosis, RAB25, KIT, SYT13, and TMEM79 were expressed in piglet jejunums and 2D organoids but not in ST cells. However, in piglet jejunums, a difference of 3 log_2_ was observed for these four genes. SYT10 was expressed only in 2D organoids. Globally, for the organoids, no variation of more than 1 log_2_ was observed between the infected and control samples (Additional file 19B).

### Comparison of innate immune responses and viral genome expression control among infected models

Functional enrichment analysis revealed that genes linked to the immune response were upregulated in ST cells (9 hpi and 24 hpi), 2D organoids (MOI of 1), and piglet jejunums (24 hpi). The log_2_-fold change and adjusted *P* value of the differential expression analysis were associated with these genes. All the genes were expressed in control samples. The 61 genes involved in innate immune responses were expressed in the ST cells, piglet jejunums, and 2D organoids, with the exception of CXCL9, IFNB1, and IL6 in the control samples (Additional file 20). In comparison with the ST cells, the 2D organoids and piglet jejunums expressed 2 log greater amounts of TRIM15, ZBP1, OAS2, OASL, IRF7, F2RL1, FGL2, CASP1 and ISG15.

**Figure 10:**
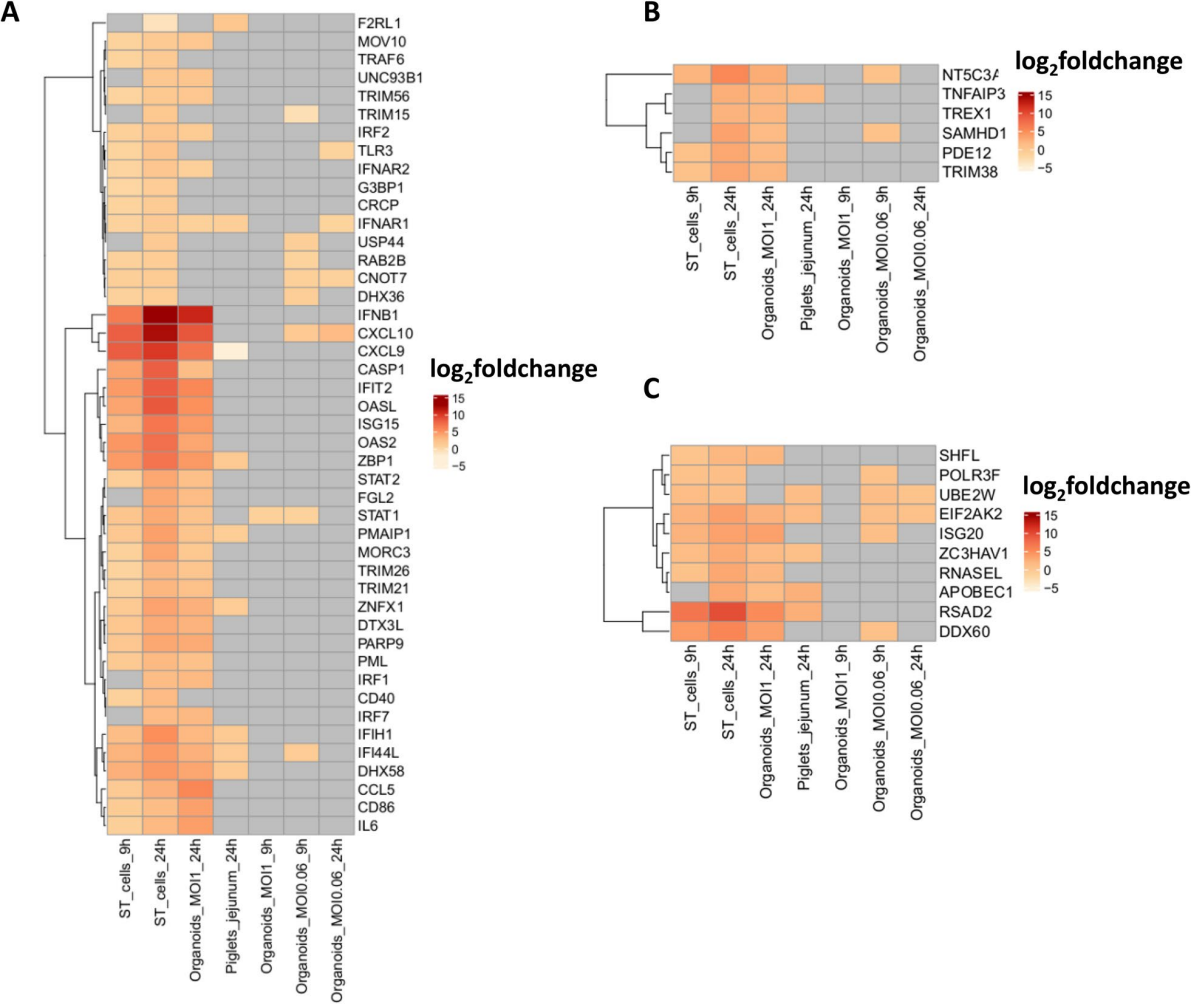
**Differential gene expression involved in innate immune responses and in the control of viral genome expression**. Genes involved in the activation of innate immune responses were retrieved from ENSEMBL. Only the genes whose adjusted *P* value was less than 0.05 in at least one of the models were included in the heatmaps. The grey colour corresponds to inconclusive data (e.g., adjusted *P* value > 0.05). The genes were then split according to their precise biological function: **A**. activation of the innate immune system, **B**. inhibition of the innate immune system, and **C**. control of viral genome expression.

These results are represented in a heatmap (Figure 10A), in which we observed that ST cells infected at an MOI of 0.06 were able to overexpress 41 and 48 genes at 9 hpi and 24 hpi, respectively. Infection of 2D organoids at an MOI of 0.06 induced the overexpression of 8 and 4 genes, which are involved in the innate immune system, after 9 h and 24 h, respectively. After 24 h of infection at an MOI of 1, the 2D organoids overexpressed 34 genes rather than a single gene at 9 hpi. Among these upregulated genes, several were common between ST cells and organoids, such as IFNB1, CXCL10, CXCL9, ISG15, OASL, IFIT2, and OAS2, with strong log_2_-fold changes (greater than 4). In addition, ST cells (9 hpi and 24 hpi) and organoids (MOI of 1 and 24 hpi) overexpressed TRIM56, TRIM26, and TRIM21. At 24 hpi, infected piglet jejunums and 2D organoids infected at an MOI of 0.06 had similar expression of CNOT7 and CXCL10. The unique gene overexpressed by all the models and all the MOIs at 24 hpi was IFNRA1. This gene was also expressed at 9 hpi in ST cells. At 24 hpi, infected piglet jejunums, ST cells, and 2D organoids infected at an MOI of 1 specifically expressed IFIH1, DHX58, IFI44L, ZNFX1, and PMAIP1. However, piglet jejunums expressed these genes at a lower level, with a maximum difference of 6 log_2_-fold change for ZBP1 in comparison with that observed in ST cells.

At 24 hpi, the ST cells and 2D organoids infected at an MOI of 1 expressed genes implicated in the inhibition of the innate immune response, such as NT5C3A, TNFAIP3, TREX1, SAMHD1, PDE12, and TRIM38 (Figure 10B). TNFAIP3 was upregulated in piglet jejunums. At 9 hpi, 2D organoids infected at an MOI of 0.06 expressed SAMHD1 and NT5C3A.

The infected models also exhibited an activation of genes involved in the control of viral genome expression (Figure 10C). Interestingly, at 24 hpi, infected piglet jejunums, ST cells, and organoids (MOI of 1) all expressed RSAD2, APOBEC1, and ZC3HAV1. At 24 hpi, the 2D organoids (MOI of 0.06), in common with the other models, exhibited the overexpression of UBE2W and EIF2AK2.

## Discussion

Alternative models for studying host–virus interactions, such as explants, organoids or organ-on-chips, are currently being developed to address the methodological and ethical problems associated with in vitro and in vivo models, respectively. The challenge for an alternative model is to be cultivable in the laboratory with the ability to reproduce the major functions of the target organ in a repeatable manner. This is why the first intestinal organoids were produced in 2009 by culturing Lgr5 adult intestinal stem cells from mice in an extracellular matrix and a medium enriched in growth factors [16]. In 2013, Gonzalez and colleagues transferred this protocol to a porcine model [15].

To evaluate the ability of the porcine intestinal organoid model to overcome the challenges of alternative models, we produced 3D organoids from the intestinal crypts of three stillborn piglets and maintained them until passage 25. To perform viral infection, 3D organoids were dissociated, and the cells were spread to produce 2D organoids, exposing the apical pole containing the viral receptors, that were subsequently infected with a TGEV strain. The ability of the model to reproduce the structure and cellular diversity and to achieve homogeneous transcript expression among three donor piglets was analysed.

Immunostaining of ZO1, a tight junction protein localized in the apical pole, confirmed the expected apical-in conformation of 3D organoids and the presence of tight junctions in 2D organoids. Immunostaining experiments and RNA-seq analysis confirmed that our 3D and 2D organoids displayed cellular diversity in the porcine intestinal epithelium. However, the expression of PYY and SI, biomarkers of enteroendocrine cells and enterocytes, was lower in 3D and 2D organoids than in piglet jejunums [32]. SI expression is induced during the developmental stage of the animal [40]. However, the difference in SI expression between organoids from suckling and weaned pigs and the organ from which they originate has already been reported by Mussard et al. [41]. We hypothesize that culture conditions, such as the composition of the medium of the organoids, do not allow the expression of the SI to be maintained. In addition, the decrease in PYY may be due to the lack of neural and humoral pathways in 3D and 2D organoids that are essential for hormone secretion [42].

To characterize the robustness of the 3D and 2D organoid models, we first analysed the variability in transcript expression among biological replicates of the experimental models by RNA-seq. After the isolation of crypts from our three donor piglets, we observed that one donor piglet had an overall transcript expression profile that was different from that of the two other pigs. However, even if we had initial variations in the crypt transcriptome, the cultivation and differentiation methods allowed the 3D organoids to display a very good correlation with their transcript expression starting at the 5^th^ passage, which was maintained at the 14^th^ and 25^th^ passages and for all donor piglets. The 3D organoids could be cultivated through long-term expansion with highly reproducible gene expression, which was maintained in 2D organoids. The 3D and 2D organoid models from the three donor piglets showed less heterogeneity than the jejunum models from the three piglets did. This observation might be due to the standardization of cell culture, the multiple passages, the composition of the culture medium and the extracellular matrix proteins, which induced an equivalent expression program in the organoids from the three different donor piglets. In vivo, the concentrations of these proteins may be lower, and gene expression can also be influenced by nutrients, such as butyrate [43], and by the composition of the microbiota [44].

The 3D and 2D organoids expressed genes involved in nutrient absorption and epithelial organization. Crypts, 3D and 2D organoids expressed genes involved in cell–cell digestive development, adhesion and lipid metabolic processes. The absorption of lipids and metabolism of fatty acids occur in the enterocytes of the intestine [45]. Experiments in organoids by Joo et al. revealed that apical-out porcine intestinal organoids were able to absorb lipid droplets, but the apical-in 3D organoid conformation was not able to do so. These results confirmed that organoids can be used to analyse lipid absorption when the issue of organoid polarization is considered [46]. Another role of the intestine is the absorption of fructose and glucose [47]. Our 3D and 2D organoids expressed genes encoding 25 sugar transporters whose expression levels were the same as those in piglet jejunums. However, we note that this observation was not valid for other members of the same family (e.g., SLC2A2 and SLC5A1). Overall, these results are in accordance with those of Hoffman et al., who demonstrated, in Transwell culture, active transport of glucose in 2D organoids [48].

However, a major difference in transcription between organoids and piglet jejunums is the expression of genes involved in extracellular matrix (ECM) organization, such as those encoding collagen or laminin. The Matrigel used to cultivate 3D and 2D organoids contains several ECM proteins, including collagen, laminin, and heparin sulfate proteoglycans [49], which mimic the extracellular matrix. As already mentioned by Van der Hee et al., the addition of Matrigel induces the downregulation of these genes in cultivated organoids [50]. Nevertheless, even when the genes encoding the constituent proteins of the ECM were downregulated, the cells were still able to activate pathways linked to the ECM, such as the TGFB1 pathway, which is mediated by LTBP3 and the transcription factor SMAD3 (Figure 5D). This pathway has been shown to control the growth of mouse colonic intestinal organoids by repressing cellular division [51].

The expression profile of 2D organoids was more similar to that of ST cells than to that of piglet jejunums. We hypothesize that this was partly due to the increased activity of genes involved in the cell cycle and DNA replication in ST cells and 2D organoids. These cells are indeed subjected to the addition of a large number of growth factors to their culture medium [36]. Moreover, these cells are constantly dividing and differentiating because of the various cell passages, which is definitely not the case for the in vivo piglet jejunums.

After characterizing our 3D and 2D organoid models, we estimated the suitability of the organoid model for studying viral infections. Changes in gene expression among TGEV-infected jejunum tissue, 2D organoids, and ST cells were compared to determine to what extent the 2D organoids reproduced the host–virus interactions that develop in jejunum tissues.

The 2D organoids, like the 5-week -old piglets and the ST cells commonly used to produce TGEV, expressed the viral receptor (pAPN) and its coreceptor (EGFR), as verified by RNA-seq. Interestingly, the Purdue strain of TGEV used in this study could infect 2D organoids at both MOIs (0.06 and 1). However, at the same MOI of 0.06, at 24 hpi, in ST cells, a 2-log greater level of viral N gene expression was detected in the supernatant and a 1-log greater level was detected in the cells compared to that in 2D organoids. Given that the ST cell line is composed of a single cell type permissive to TGEV, viral production is faster in this cell line than in the pluricellular 2D organoids. Nevertheless, after 24 hpi, in the supernatant, the same level of viral production (3 log compared with 0 h) by ST cells infected at an MOI of 0.06 was also detected in organoids infected at an MOI of 1. In comparison with published data, our 2D organoids infected at an MOI of 1 have the same dynamic of replication over time as the H165 vaccine and attenuated strain of TGEV, with an increase in the viral N gene inside the cells in porcine ileum organoids [52]. These results differ from those of the study by Yin et al., who analysed the infection of porcine jejunum organoids with the TGEV AHHF strain, a pathogenic and natural recombinant strain of Purdue and Miller clusters, at an MOI of 1 and reported that viral RNA decreased after 24 hpi in the cells [20]. In parallel, we tested infection with a pathogenic TGEV strain belonging to the Miller cluster, and the infection dynamics were comparable to those reported for the TGEV AHHF strain in the cellular compartment of the organoids (data not shown). These results suggest markedly different infection patterns between the Purdue and Miller strains, which could indicate that organoids are easier to infect with lab strains (such as Purdue) than with field strains (such as Miller), even when the field strain is more pathogenic. To improve the infection of field strains of viruses, the addition of trypsin or bile may be considered, as is the case for sapovirus infection of human intestinal organoids [53].

The intestinal epithelium functions as an epithelial barrier that protects against pathogens. The 2D organoids expressed genes encoding tight junction proteins, claudins (CLDN) 3/4/7/9/15/23, cadherins 1/17/24, ZO1, TJ2/3, and occludin. Claudin and occludin proteins allow the diffusion of cations from the lumen to the basal membrane [54]. Together, cadherin and tight junction proteins ensure cellular adhesion and block pathogen entry [55]. However, the tight junction proteins CLDN3/7/23 are expressed in pig jejunums, unlike in ST cells. Interestingly, TGEV infection of 2D organoids and piglet jejunums induced a decrease in the expression of CLDN3, a key regulator of intestinal tight junctions [56]. Taken together, our results indicate that 2D organoids represent an appropriate model for studying the effect of viral infection on the epithelial barrier.

TGEV infection induced gene expression changes in ST cells, 2D organoids and piglet jejunums. We observed more deregulation in infected ST cells than in infected 2D organoids and piglet jejunums (ten and three times more differentially expressed genes, respectively, at 24 hpi). We hypothesize that these differences are due to the higher cellular diversity in 2D organoids and piglet jejunums, which also contain cells not directly targeted by the virus and therefore are less affected by the infection. Moreover, we hypothesize that the culture conditions of the organoids, with the dissociation and culture in Matrigel, could induce the expression of genes involved in the innate immune response without infection. In fact, in our study, compared with ST cells, uninfected 2D organoids expressed 2 log greater amounts of ZBP1, OASL, OAS2 and TRIM15. This higher basal expression level might partially mask the response induced by TGEV infection. In addition, at an equivalent MOI, the viral N gene was detected more rapidly and strongly in the ST cells than in the organoids, suggesting faster gene activation in response to infection. Furthermore, piglet jejunums are not composed exclusively of intestinal epithelium but notably include the lamina propria and other mucosa containing adaptive immune cells, which could fight TGEV infection [57].

B cells and T cells localized in the lamina propria are crucial for immune responses against enteric viruses. In fact, antibodies produced by B cells play a crucial role in neutralizing viruses in the mucus layer, whereas T cells clear enteric viruses [58]. However, our 3D and 2D organoids lacked lamina propria. To overcome these problems, cocultures of intestinal organoids and lymphocytes have been developed in mice, which would be worth transferring to pig models [59].

In our study, we verified the ability of 2D organoids to express genes of the WNT and NOTCH pathways, which control the cellular diversity of the intestinal epithelium [38, 52]. TGEV is known to disrupt the self-renewal of stem cells in porcine ileum organoids through the WNT signalling pathway with the H165 strain belonging to the Miller cluster at an MOI of 1 [52]. However, in our study, we observed no change in the activity of this signalling pathway in our 2D jejunum organoids infected with a TGEV strain belonging to the Purdue cluster (at an MOI of 0.06 or 1) compared with their controls. We hypothesized that this difference may be due to biomolecular differences between the extracted tissues and/or the different TGEV strains used.

Our study revealed that host–virus interactions developed in 2D organoids at two MOIs. The expression of genes involved in nucleoside trisphosphate biosynthesis, which is essential for the synthesis of cellular DNA and RNA [60], was upregulated in 2D organoids and piglet jejunums (24 hpi at an MOI of 0.06). In ST cells, this function was enriched only at 9 h post infection. This function is essential for the replication of the viral genome through the hijacking of host nucleotides [60]. Studies have been conducted to analyse triphosphate analogues as antiviral drugs against SARS-CoV-2 in Syrian golden hamsters [61]. Our study supports the relevance of porcine 2D organoids in the analysis of the nucleotide triphosphate biosynthesis pathway as an antiviral target.

Upon infection, all our models showed upregulated genes involved in the viral defence response, particularly those involved in the activation of the innate immune system. Interestingly, in 2D organoids infected at an MOI of 0.06 (9 hpi and 24 hpi), only a few genes were activated, such as receptors targeting viral dsRNA (TLR3) or those genes involved in the initiation of the IFN production pathway, including CNOT7, CXCL10, and STAT1 [62, 63]. 2D organoids, infected with a lower MOI, offer the possibility to decipher the first step of the antiviral responses implicated in TGEV infection, in contrast to ST cells, which overexpressed more than 40 genes at 9 hpi. At 24 hpi, all models expressed genes involved in the IFN pathway, such as the first subunit of the IFN-I receptor, IFNRA1, and IFI44L, an interferon-stimulated gene (ISG) with antiviral function, and feedback control of IFN production [64, 65]. In addition, we observed the expression of ZBP1 and ZNFX1, which are dsRNA-detecting proteins that induce type I interferon (IFN-I) production, only 24 hpi [66, 67]. Their expression was also reported by Yin et al., who infected porcine intestinal organoids with the AHHF strain of TGEV [20]. Our study demonstrated that 2D porcine intestinal organoids can be exploited to dissect the innate immune response, including the IFN pathway.

Three of these experimental models (ST cells, piglet jejunums and 2D organoids) were shown to overexpress apolipoprotein B mRNA editing catalytic polypeptide-like 1 (APOBEC1) during infection. The APOBEC protein family includes proteins involved in RNA editing (the modification of C to U) [68]. APOBEC 2 and APOBEC 4 are poorly studied, unlike APOBEC3, which is involved in the restriction of retroviral infection by human immunodeficiency virus 1 (HIV-1) [69]. None of these proteins were detected under either control or infection conditions. APOBEC1 was initially characterized as playing a role in cholesterol maintenance and lipid metabolism and is associated with cancer [70]. However, APOBEC1 is also known to modify the HIV-1 genome and reduce infectivity in 293 T cells [71]. Another study revealed that APOBEC1 can modify the SARS CoV-2 genome in intestinal cells [72]. APOBEC1 is highly expressed in the human small intestine; however, its expression in pig models is not well characterized [70]. Its expression in all infected models raises the question of its implications for porcine enteric virus infection. Porcine intestinal organoids thus offer the possibility of analysing the activity of APOBEC1 against enteric viruses such as TGEV.

The infected models upregulated genes encoding proteins responsible for the restriction of viral genome replication, such as UBE2W, EIF2AK2, and ZC3HAV1. UBE2W is a ubiquitin-conjugating enzyme that is involved in the inhibition of viral reverse transcription induced by TRIM5α [73]. However, UBE2W is also involved in the ubiquitination of TRIM21, which is essential for its ability to activate the NF-KB pathway and was upregulated in all the models in our study. ZC3HAV1 is known to be an ISG that can bind to CpG-rich viral RNA to recruit nucleases, as observed in porcine epidemic diarrhoea virus (PEDV), another porcine alphacoronavirus [73–75]. Although the role of the ISG EIF2AK2 in swine infection remains elusive, it has been shown to activate cytokine production during SARS-CoV-2 infection [76]. 2D organoids appear to be candidate experimental models for understanding the involvement of restriction proteins in TGEV viral replication induced by the IFN pathway.

In conclusion, piglet intestinal organoids represent a model of the interface between cell lines and animals, which offers the possibility of deciphering the multiple functions of the intestinal epithelium and enteric infection processes. They reduce the need to use animals for a detailed study of host‒pathogen interactions to respond to emerging viruses that affect the pig industry.

## Supplementary Information

**Additional file 1. Comparison of gene annotations among pig, human and mouse species**. Table representing the number, percentage and average number of annotated genes among the pig, human and mouse species.

**Additional file 2. Characterization of 3D and 2D organoids**. A. Pictures representing the different steps of 3D organoid production and maintenance during 25 passages. Scale bar =100 µm. B, C. Images of 2D organoid immunostaining with different antibodies observed with an Olympus CKX41 fluorescence microscope. ZO1: apical Pole (B) and villin 1 at 40X magnification (C). D. Heatmap and hierarchical clustering of the log base e (1+FPKM) values of transcripts encoding a selection of biomarkers associated with the different cell types of the intestinal epithelium.

**Additional file 3. List of the biomarker genes of intestinal epithelial cells.** Table representing the biomarkers used in this study and the accession numbers of their transcripts. For each biomarker, a reference is included.

**Additional file 4. Expression of solute carrier proteins (SLCs) in piglet jejunums, crypts, 3D organoids at passages 5, 14 and 25, 2D organoids and ST cells**. The average of DESeq2-normalized counts transformed into log base e of 1+normalized counts was calculated. A grey box indicates that the adjusted *P* value of the gene is greater than 0.05.

**Additional file 5. Table of log(1+FPKM) values of the transcripts encoding solute carrier proteins (SLCs) in piglet jejunums, crypts, 3D organoids at passages 5, 14 and 25, 2D organoids and ST cells**. The average of the log base e of 1+FPKM values was calculated for each model.

**Additional file 6. Table of the log**_**2**_ **(FPKM) values calculated for each noninfected experimental model.** For each model—ST cells, piglet jejunums, 3D organoids (5^th^, 14^th^ and 25^th^ passages) and 2D organoids—the FPKM values were calculated and are displayed in this table.

**Additional file 7. The determination coefficient (R**^**2**^) **values were calculated on the basis of the DESeq2 normalized counts transformed into log**_**2**_**(1+normalized counts) and log**_**2**_**(1+FPKM) values.** To compare gene and transcript expression, the R² between the respective R^2^ values was calculated and is represented in the graphic

**Additional file 8. Comparison of transcript expression among piglet jejunums, crypts, 3D organoids, 2D organoids and ST cells**. The average of the log_2_(FPKM) values was calculated, and for each experimental model, log_2_(FPKM) values were sorted on the basis of log_2_(FPKM)>1 and padj<0.05. The determination coefficient (R²) values were calculated between the log_2_(FPKM) values of the experimental models.

**Additional file 9. Table of the Gene Ontology analysis results affiliated with each comparison between uninfected experimental models**. After the calculation of log_2_(FPKM) values for each noninfected experimental model, a Gene Ontology analysis was performed for the shared and specific transcripts. The corresponding enrichment tables are displayed in each sheet of the table.

**Additional file 10. Analysis of the function of genes expressed in the experimental models.** A. Gene Ontology (biological process) analysis of the transcripts with a log_2_(FPKM) >1 and a padj<0.05 that were shared by crypts, piglet jejunums, 3D and 2D organoids and ST cells. B. Gene Ontology (molecular function) analysis of the transcripts with a log_2_(FPKM) >1 and a padj<0.05 and expressed by piglet jejunums, crypts, and 3D and 2D organoids. C. Gene Ontology (biological process) analysis of the transcripts with a log_2_(FPKM) >1 and a padj<0.05 and shared by piglet jejunums, 3D, and 2D organoids. D. Gene Ontology (biological process) analysis of the transcripts with a log_2_(FPKM) >1 and a padj<0.05 and expressed by ST cells and 3D and 2D organoids. E. Gene Ontology (biological process) analysis of the transcripts with a log_2_(FPKM) >1 and a padj<0.05 and expressed by 3D organoids after 25 passages. F. Gene Ontology (biological process) analysis of the transcripts with a log_2_(FPKM) >1 and a padj<0.05 and expressed by ST cells.

**Additional file 11. Functional analysis of gene expression for the different experimental models with human annotations.** Functional enrichment was performed with the molecular function tree of Gene Ontology and by considering only the transcripts whose log-scale FPKM was greater than 1. Functional terms associated with an adjusted *P* value greater than 0.05 were discarded. This additional file is the equivalent of the analysis performed with the mouse annotations presented in Figure 5. A. Functional enrichment of the genes associated with the transcripts expressed in crypts, piglet jejunums, and 3D and 2D organoids. Similar Gene Ontology terms were grouped into more generic functional categories (corresponding to different colours). B. Functional enrichment of the genes associated with the transcripts expressed in 2D organoids and ST cells. C. Functional enrichment of the genes associated with the transcripts expressed only in piglet jejunums.

**Additional file 12. Table representing the log**_**2**_**-fold changes in genes implicated in the morphology of the epithelium.** The log_2_-fold changes in genes implicated in the morphology of the intestinal epithelium were calculated between 3D organoids at the 25^th^ passage, 2D organoids or crypts and piglet jejunums. The values are displayed in this table.

**Additional file 13. Analysis of the expression of viral receptors**. A. Detection of aminopeptidase N by fluorescence microscopy in 2D organoids. On the left: secondary antibody alone; on the right: primary antibody against APN protein and secondary antibody. Scale bar = 200 µm B. The average of DESeq2-normalized counts transformed into logs was calculated. C. Expression of viral receptors, aminopeptidase N (APN), epidermal growth factor receptor (EGFR), and Cytidine Monophospho-N-Acetylneuraminic Acid Hydroxylase (sialic acid biosynthesis).

**Additional file 14. Detection of the viral N gene by RT‒qPCR in the intestinal tract and respiratory complex.** Cellular RNA was collected from intestinal A. and respiratory B. samples at different time points and analysed by RT‒qPCR targeting the viral N gene. *,* P* < 0.05.

**Additional file 15. Principal component analysis of ST cells and piglet jejunums for the control and infected conditions**. A. Principal component analysis based on the read counts of the infected and control ST cells. B. Principal component analysis based on the read counts of the infected and control piglet jejunums.

**Additional file 16. Gene Ontology analysis results for each infected experimental model.** The log_2_-fold change was calculated for each experimental model (ST cells, piglet jejunums and 2D organoids) between control and infected conditions. A gene was considered to be differentially expressed when its adjusted *P* value was less than 0.05 and its log_2_-fold change was either greater than 1 (upregulated) or less than -1 (downregulated). After classification, a Gene Ontology analysis was performed for these genes, and the results are displayed in this table, with each sheet representing the analysis of a different gene set.

**Additional file 17. Functional enrichment of the upregulated genes of each infected experimental model with human annotations**. The 9 hpi time point for the infected 2D organoids was not represented, as no enrichment was detected. This additional file is the equivalent of the analysis performed with the mouse annotations presented in Figure 8B

**Additional file 18. Gene Ontology analysis of the downregulated genes of the infected experimental models**. The results from 9 hpi in ST cells and 2D organoids and 24 hpi in 2D organoids at MOIs of 0.06 and 1 were not represented, as no enrichment was detected.

**Additional file 19. Gene expression implicated in endocytosis and exocytosis processes in control and infected models**. The average of DESeq2-normalized counts transformed into logs was calculated. Expression of genes involved in endocytosis (A) and exocytosis (B) in the control and infected experimental models, represented as heatmaps.

**Additional file 20. Expression of genes implicated in innate immunity and the control of gene expression in noninfected models.** The average of DESeq2-normalized counts transformed into logs was calculated for the control condition of the ST cells, piglet jejunums and 2D organoids. The first and second sheets display the activation and inhibition of the immune innate system, respectively. The third sheet presents the values for the genes involved in the control of gene expression.

## Data Availability

The RNA-seq dataset generated for this study is available on ArrayExpress under the accession number E-MTAB-15113. The genome sequence of the TGEV strain used for this study is available at the ENA under the accession number PRJEB88925.
