# Supplementary figures and images for: Relevance of porcine intestinal organoids as a surrogate for animal experimentation: application to the investigation of host–virus interactions during porcine coronavirus infection
Source: Vet Res. 2025 Nov 21;56:221. doi: 10.1186/s13567-025-01657-y (PMC12639656; doi:10.1186/s13567-025-01657-y)

## Slide 1
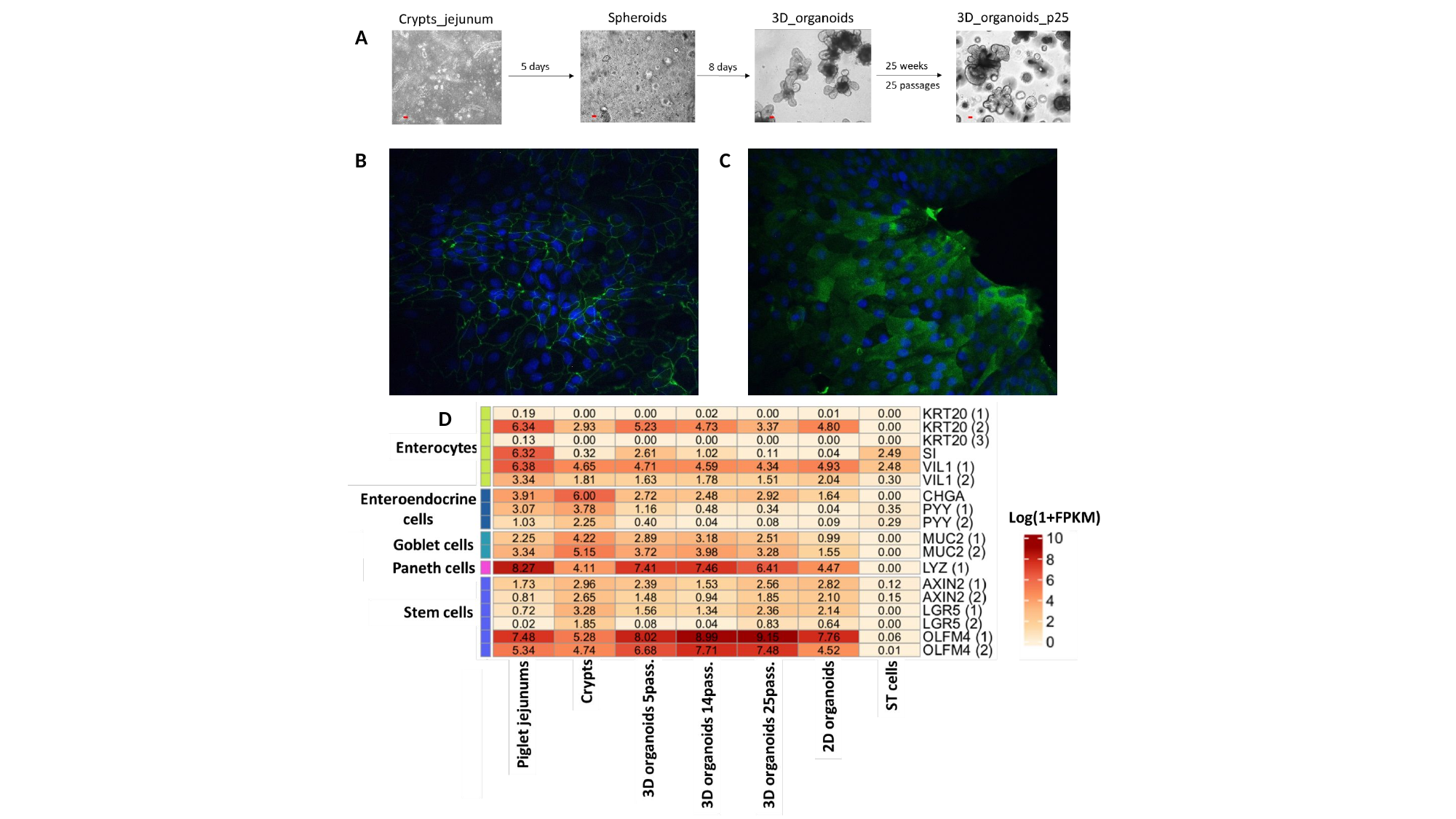

B
C
D
A

Supplement: Supplementary file 2 — Additional file 2. Characterization of 3D and 2D organoids. A. Pictures representing the different steps of 3D organoid production and maintenance during 25 passages. Scale bar =100 µm. B, C. Images of 2D organoid immunostaining with different antibodies observed with an Olympus CKX41 fluorescence microscope. ZO1: apical Pole (B) and villin 1 at 40X magnification (C). D. Heatmap and hierarchical clustering of the log base e (1+FPKM) values of transcripts encoding a selection of biomarkers associated with the different cell types of the intestinal epithelium. [file 13567_2025_1657_MOESM2_ESM.pptx]

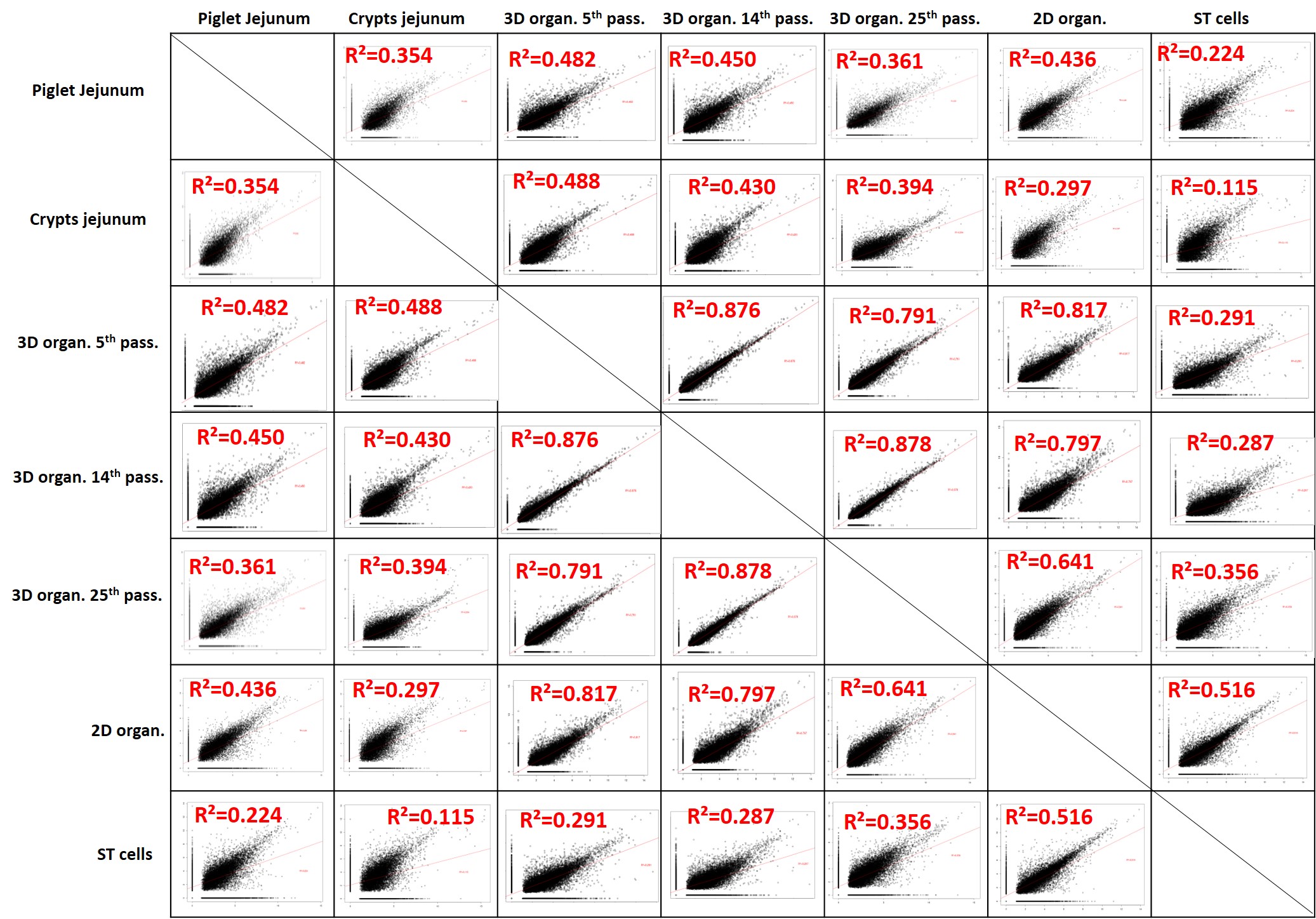

Supplement: Supplementary file 8 — Additional file 8. Comparison of transcript expression among piglet jejunums, crypts, 3D organoids, 2D organoids and ST cells. The average of the log2(FPKM) values was calculated, and for each experimental model, log2(FPKM) values were sorted on the basis of log2(FPKM)>1 and padj<0.05. The determination coefficient (R²) values were calculated between the log2(FPKM) values of the experimental models. [file 13567_2025_1657_MOESM8_ESM.jpg]

## Slide 1
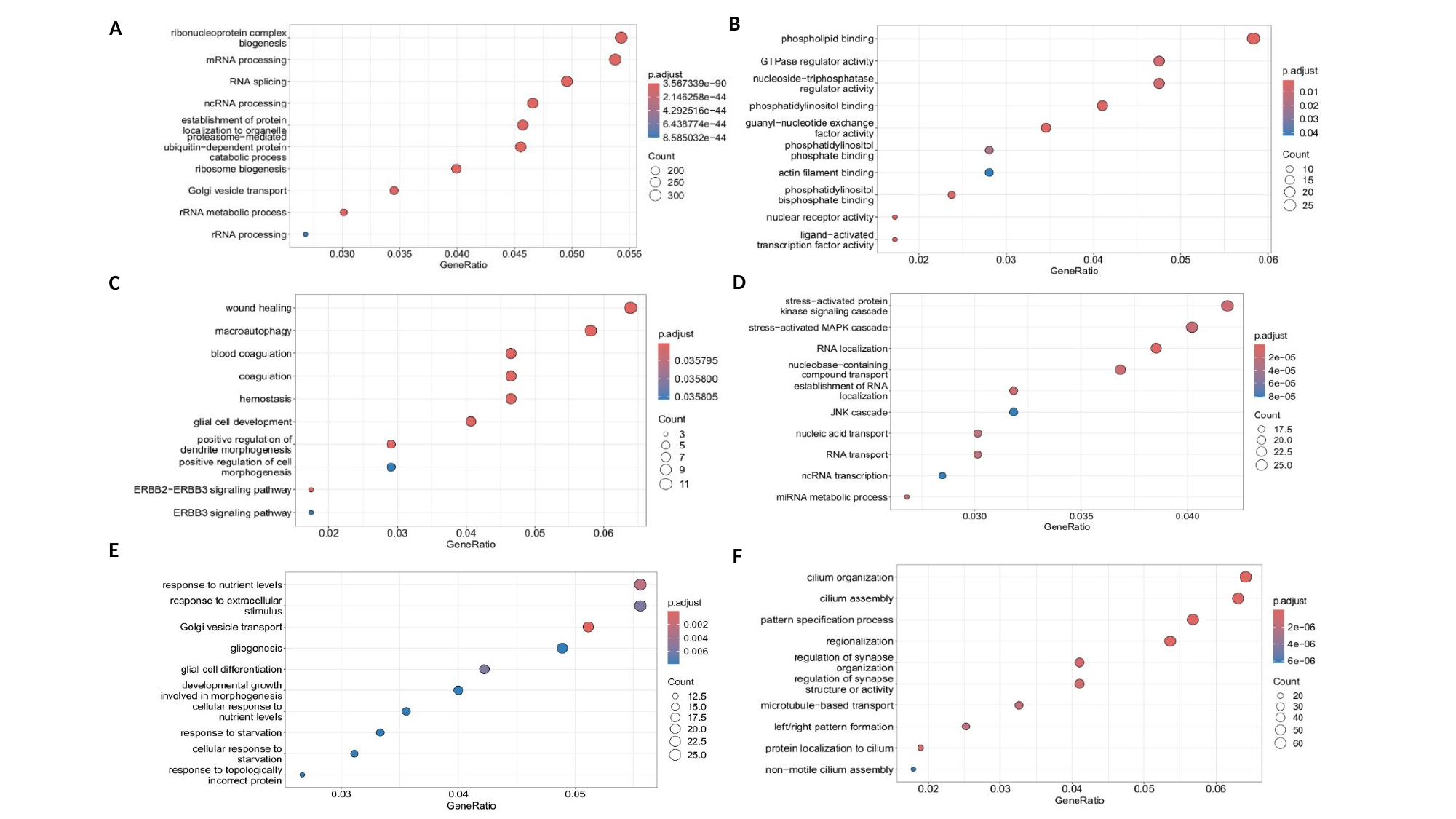

B
A
E
D
C
F

Supplement: Supplementary file 10 — Additional file 10. Analysis of the function of genes expressed in the experimental models. A. Gene Ontology (biological process) analysis of the transcripts with a log2(FPKM) >1 and a padj<0.05 that were shared by crypts, piglet jejunums, 3D and 2D organoids and ST cells. B. Gene Ontology (molecular function) analysis of the transcripts with a log2(FPKM) >1 and a padj<0.05 and expressed by piglet jejunums, crypts, and 3D and 2D organoids. C. Gene Ontology (biological process) analysis of the transcripts with a log2(FPKM) >1 and a padj<0.05 and shared by piglet jejunums, 3D, and 2D organoids. D. Gene Ontology (biological process) analysis of the transcripts with a log2(FPKM) >1 and a padj<0.05 and expressed by ST cells and 3D and 2D organoids. E. Gene Ontology (biological process) analysis of the transcripts with a log2(FPKM) >1 and a padj<0.05 and expressed by 3D organoids after 25 passages. F. Gene Ontology (biological process) analysis of the transcripts with a log2(FPKM) >1 and a padj<0.05 and expressed by ST cells. [file 13567_2025_1657_MOESM10_ESM.pptx]

## Slide 1
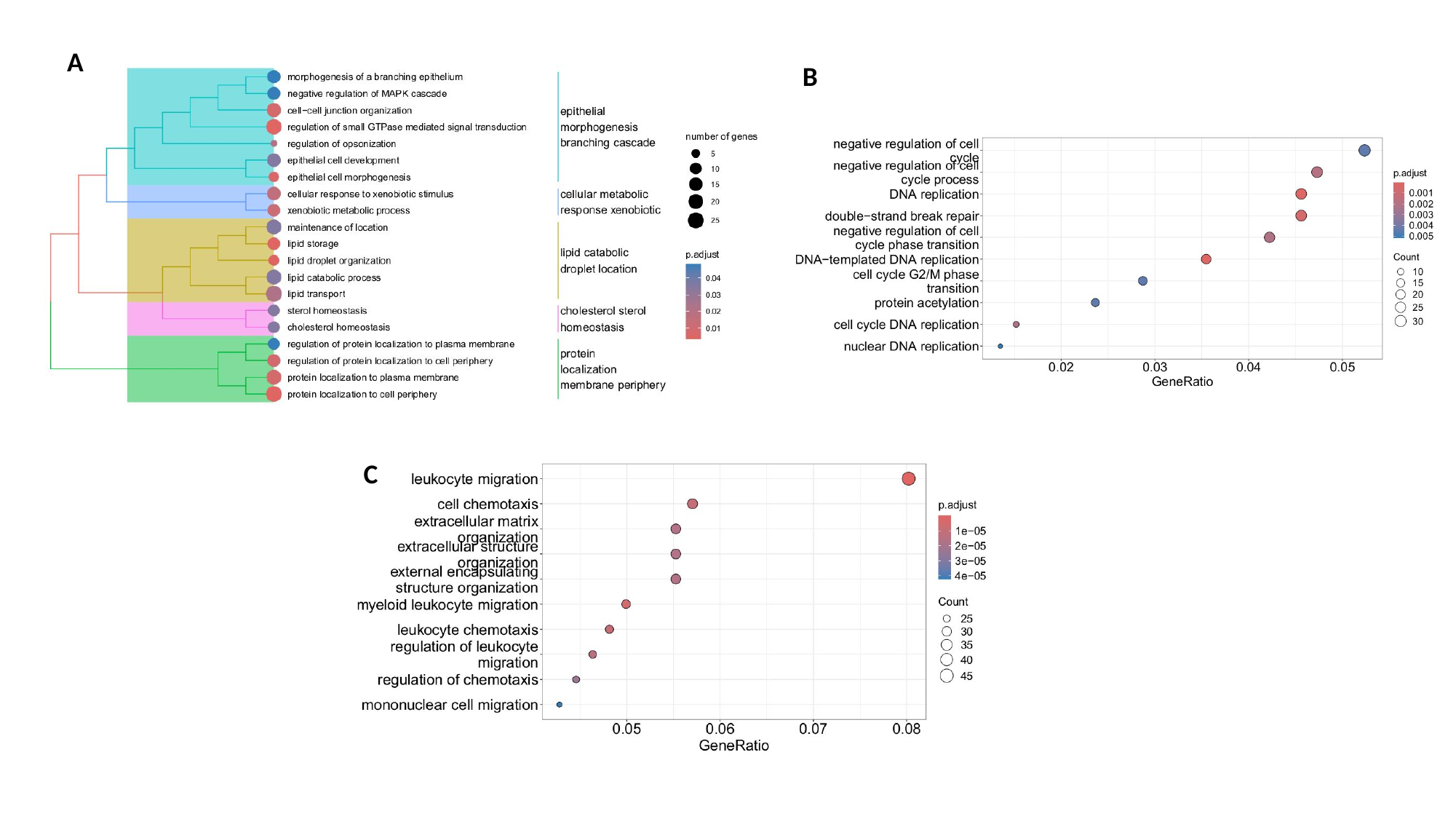

A
B
C

Supplement: Supplementary file 11 — Additional file 11. Functional analysis of gene expression for the different experimental models with human annotations. Functional enrichment was performed with the molecular function tree of Gene Ontology and by considering only the transcripts whose log-scale FPKM was greater than 1. Functional terms associated with an adjusted P value greater than 0.05 were discarded. This additional file is the equivalent of the analysis performed with the mouse annotations presented in Figure 5. A. Functional enrichment of the genes associated with the transcripts expressed in crypts, piglet jejunums, and 3D and 2D organoids. Similar Gene Ontology terms were grouped into more generic functional categories (corresponding to different colours). B. Functional enrichment of the genes associated with the transcripts expressed in 2D organoids and ST cells. C. Functional enrichment of the genes associated with the transcripts expressed only in piglet jejunums. [file 13567_2025_1657_MOESM11_ESM.pptx]

## Slide 1
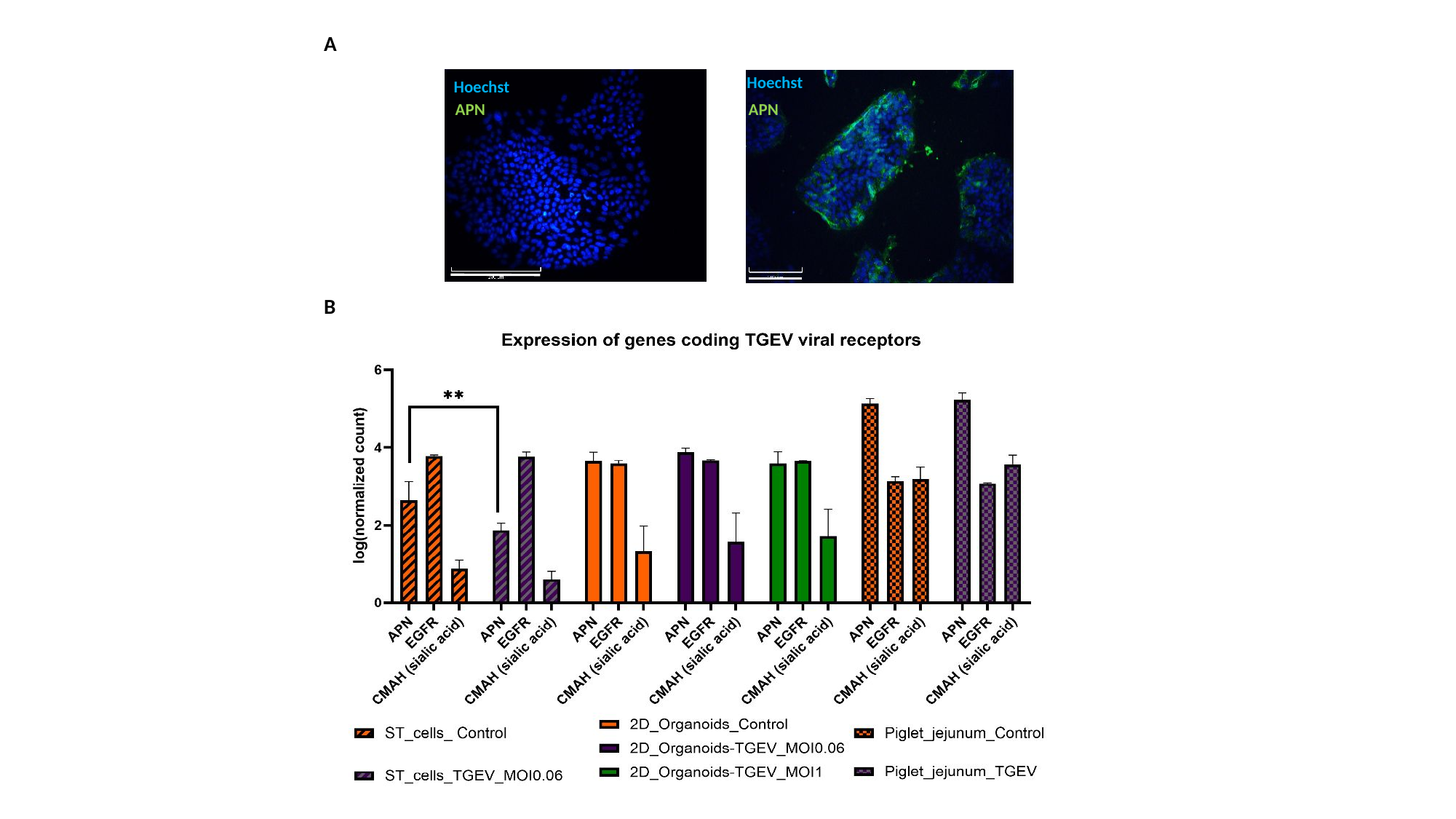

A
Hoechst
APN
Hoechst
APN
B

Supplement: Supplementary file 13 — Additional file 13. Analysis of the expression of viral receptors. A. Detection of aminopeptidase N by fluorescence microscopy in 2D organoids. On the left: secondary antibody alone; on the right: primary antibody against APN protein and secondary antibody. Scale bar = 200 µm B. The average of DESeq2-normalized counts transformed into logs was calculated. C. Expression of viral receptors, aminopeptidase N (APN), epidermal growth factor receptor (EGFR), and Cytidine Monophospho-N-Acetylneuraminic Acid Hydroxylase (sialic acid biosynthesis). [file 13567_2025_1657_MOESM13_ESM.pptx]

## Slide 1
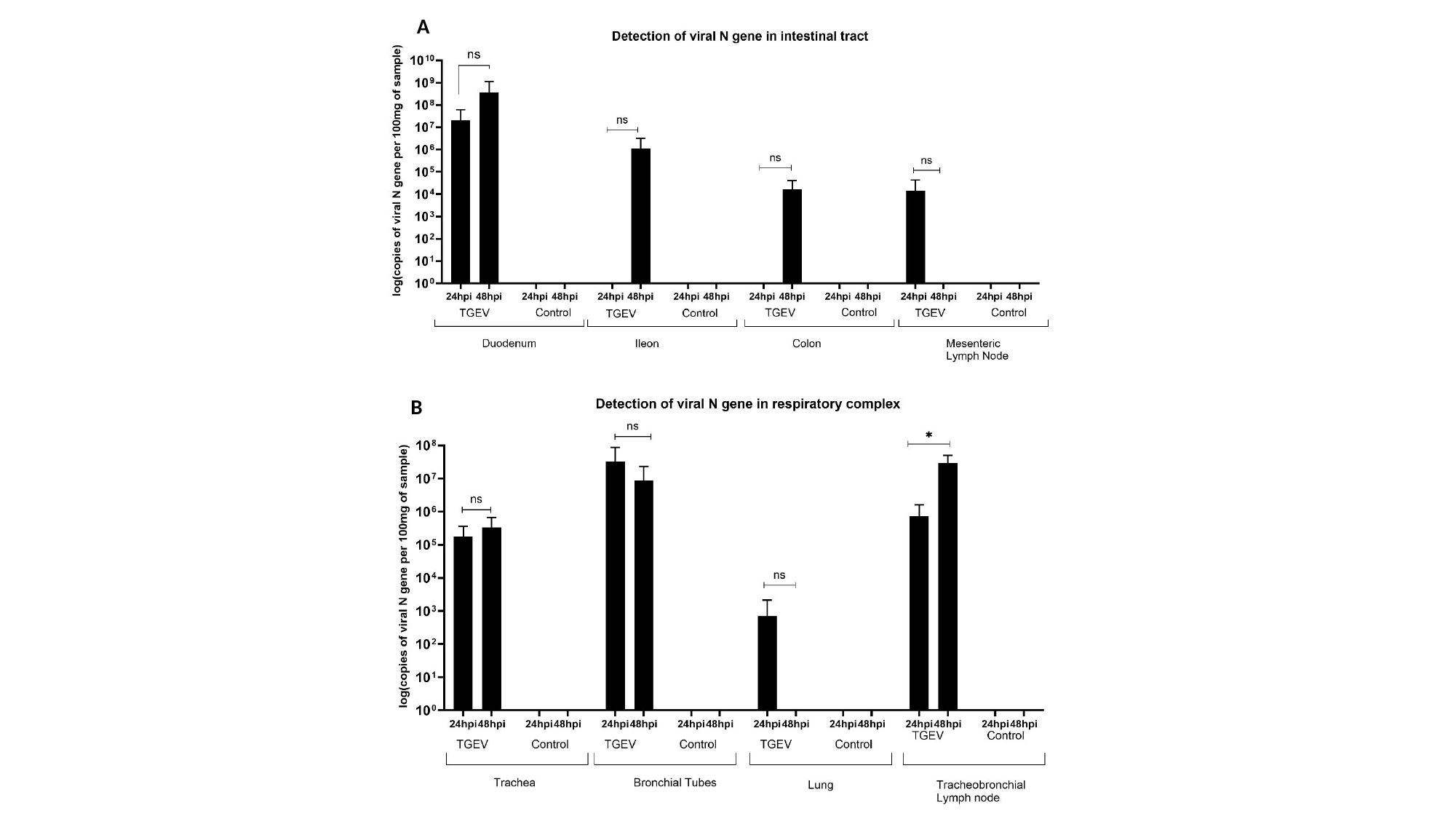

A
B

Supplement: Supplementary file 14 — Additional file 14. Detection of the viral N gene by RT‒qPCR in the intestinal tract and respiratory complex. Cellular RNA was collected from intestinal A. and respiratory B. samples at different time points and analysed by RT‒qPCR targeting the viral N gene. *, P < 0.05. [file 13567_2025_1657_MOESM14_ESM.pptx]

## Slide 1
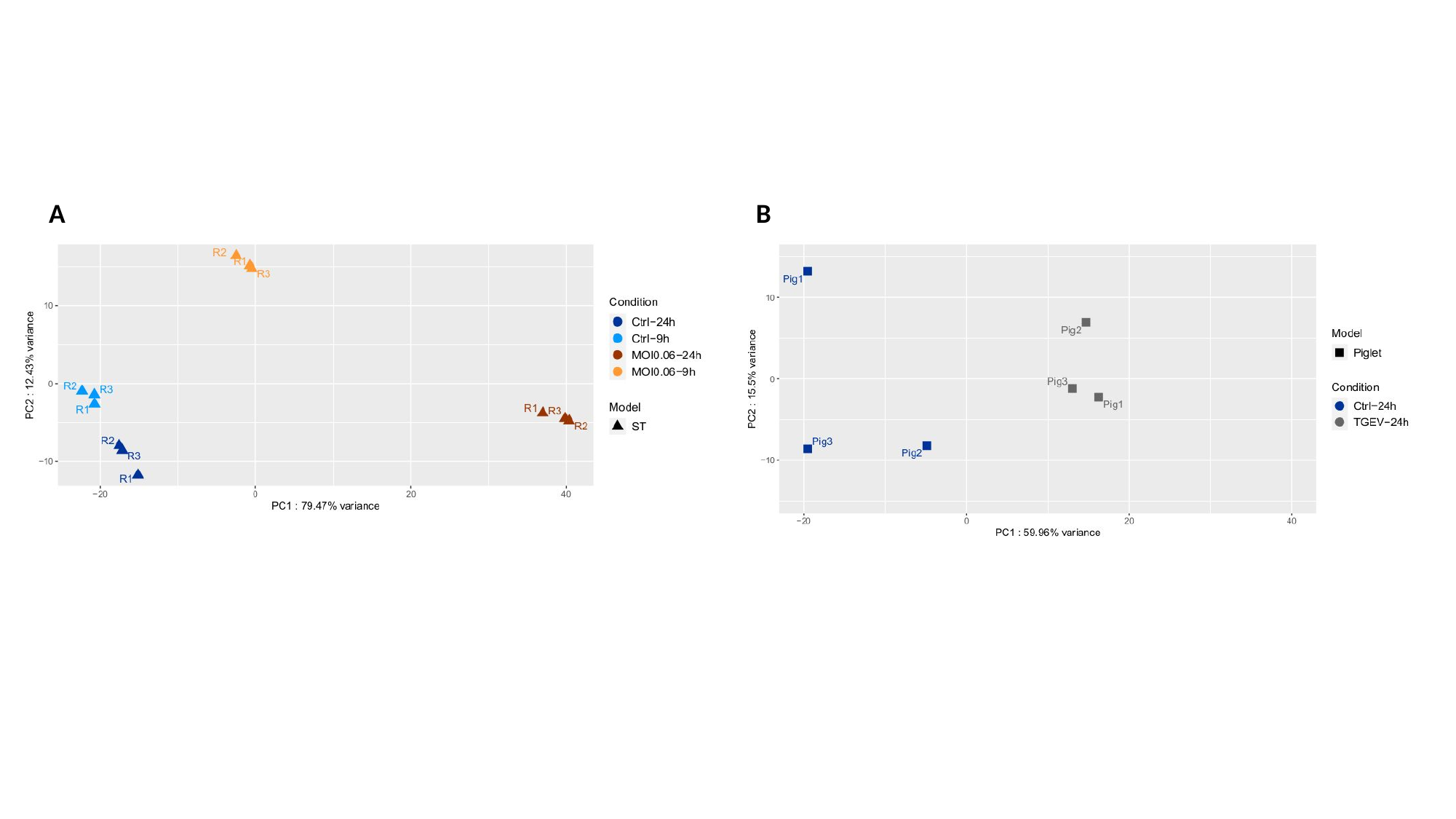

A
B

Supplement: Supplementary file 15 — Additional file 15. Principal component analysis of ST cells and piglet jejunums for the control and infected conditions. A. Principal component analysis based on the read counts of the infected and control ST cells. B. Principal component analysis based on the read counts of the infected and control piglet jejunums. [file 13567_2025_1657_MOESM15_ESM.pptx]

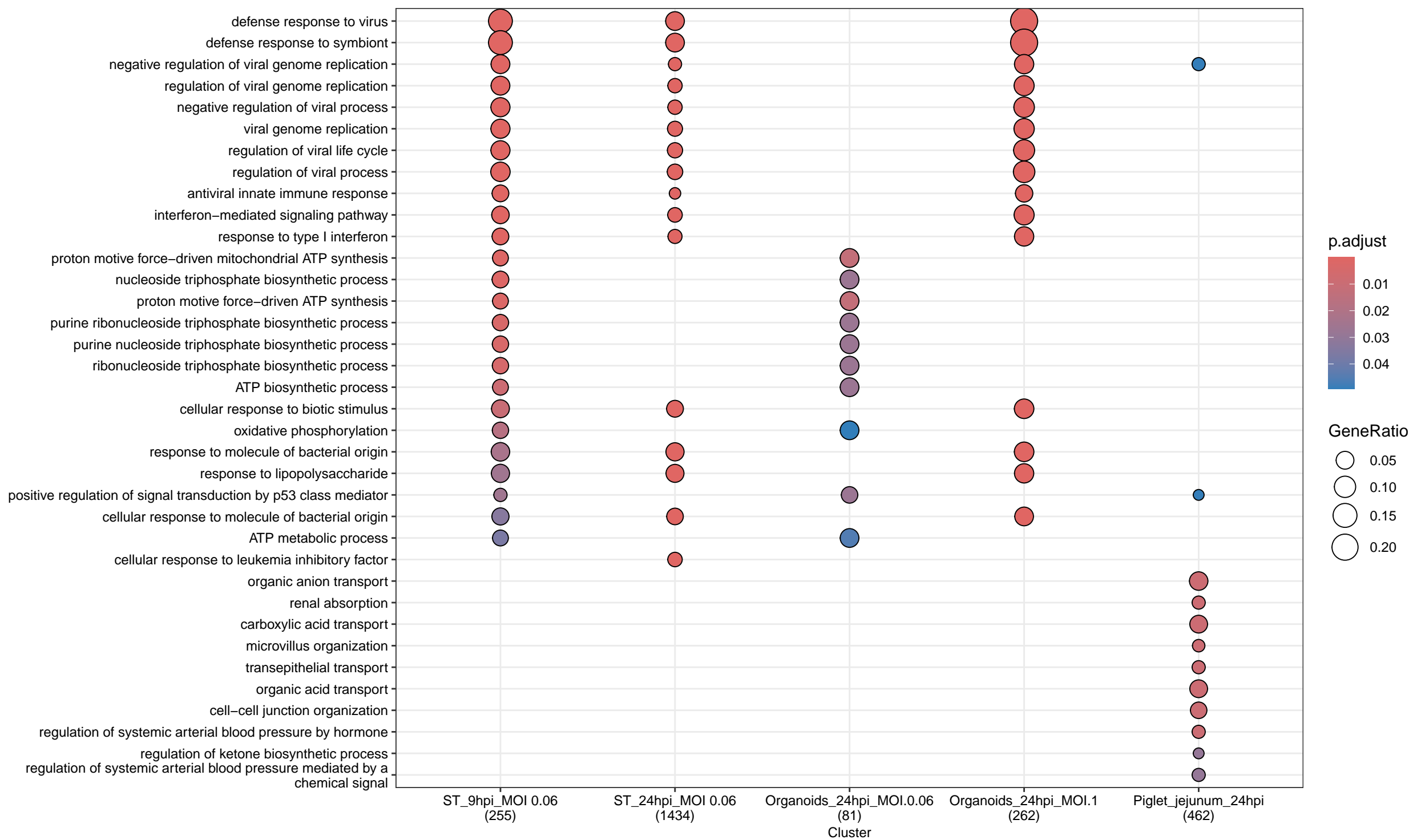

Supplement: Supplementary file 17 — Additional file 17. Functional enrichment of the upregulated genes of each infected experimental model with human annotations. The 9 hpi time point for the infected 2D organoids was not represented, as no enrichment was detected. This additional file is the equivalent of the analysis performed with the mouse annotations presented in Figure 8B [file 13567_2025_1657_MOESM17_ESM.pdf]

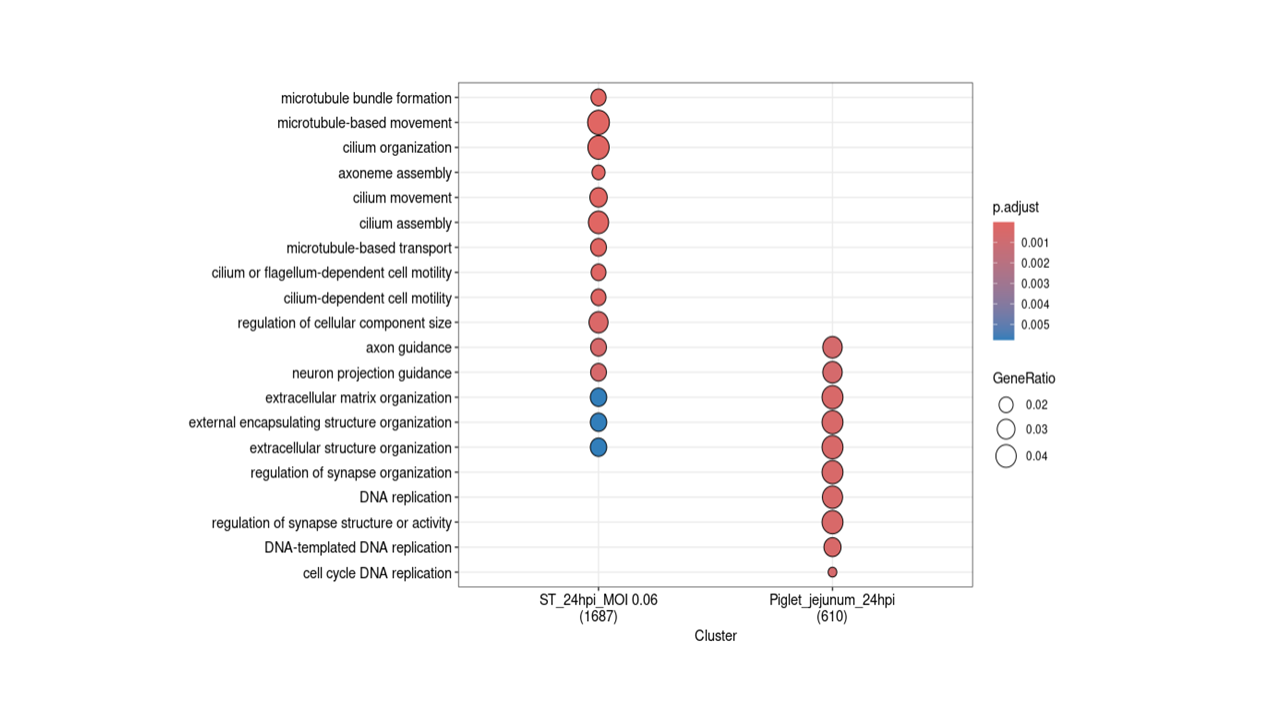

Supplement: Supplementary file 18 — Additional file 18. Gene Ontology analysis of the downregulated genes of the infected experimental models. The results from 9 hpi in ST cells and 2D organoids and 24 hpi in 2D organoids at MOIs of 0.06 and 1 were not represented, as no enrichment was detected. [file 13567_2025_1657_MOESM18_ESM.tif]

## Slide 1
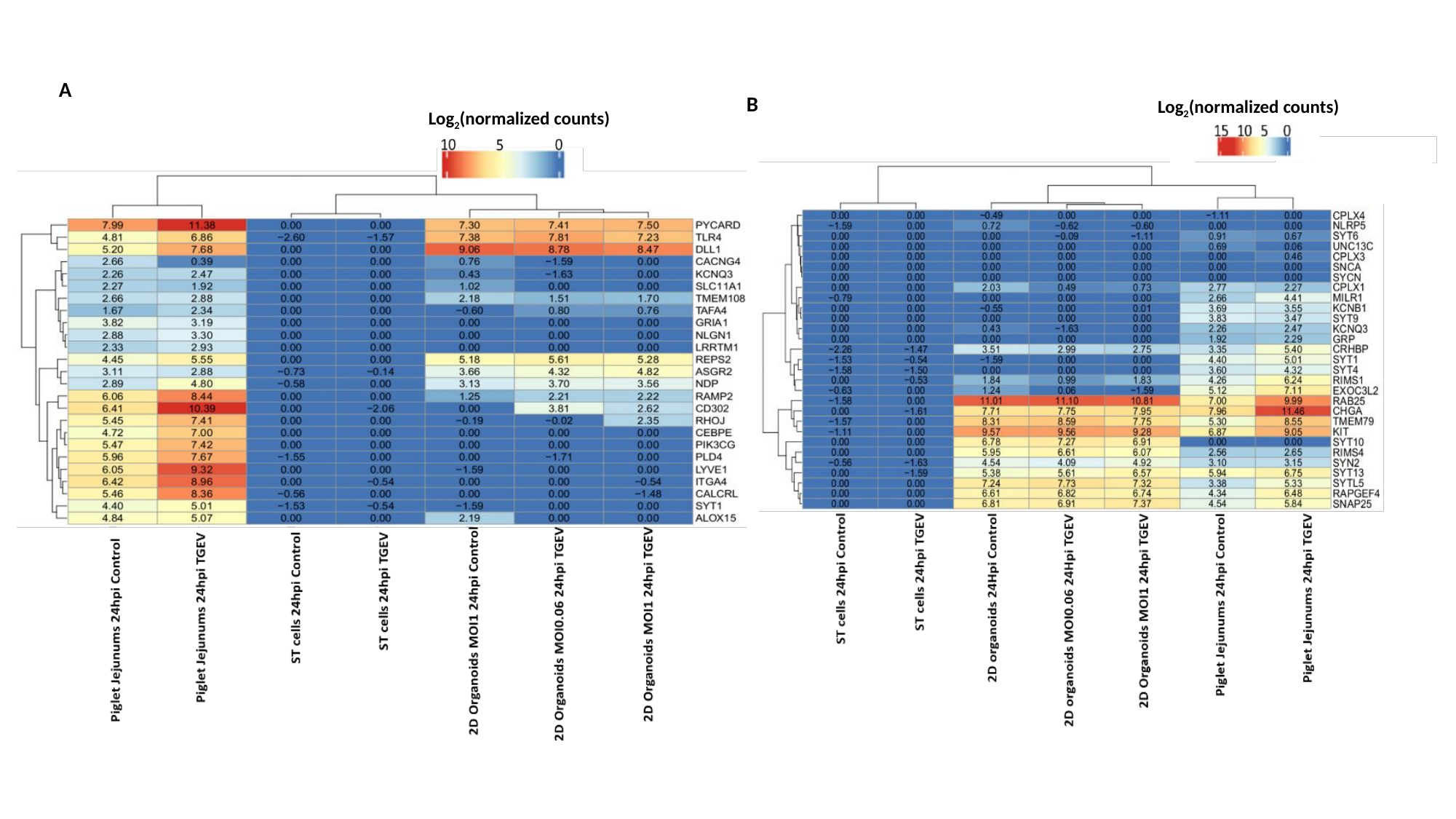

A
B
Log2(normalized counts)
Log2(normalized counts)

Supplement: Supplementary file 19 — Additional file 19. Gene expression implicated in endocytosis and exocytosis processes in control and infected models. The average of DESeq2-normalized counts transformed into logs was calculated. Expression of genes involved in endocytosis (A) and exocytosis (B) in the control and infected experimental models, represented as heatmaps. [file 13567_2025_1657_MOESM19_ESM.pptx]
