# Supplementary material for: Relevance of porcine intestinal organoids as a surrogate for animal experimentation: application to the investigation of host–virus interactions during porcine coronavirus infection
Source: Vet Res. 2025 Nov 21;56:221. doi: 10.1186/s13567-025-01657-y (PMC12639656; doi:10.1186/s13567-025-01657-y)
Supplement: Supplementary file 4 — Additional file 4. Expression of solute carrier proteins (SLCs) in piglet jejunums, crypts, 3D organoids at passages 5, 14 and 25, 2D organoids and ST cells. The average of DESeq2-normalized counts transformed into log base e of 1+normalized counts was calculated. A grey box indicates that the adjusted P value of the gene is greater than 0.05. [file 13567_2025_1657_MOESM4_ESM.pptx]

## Slide 1
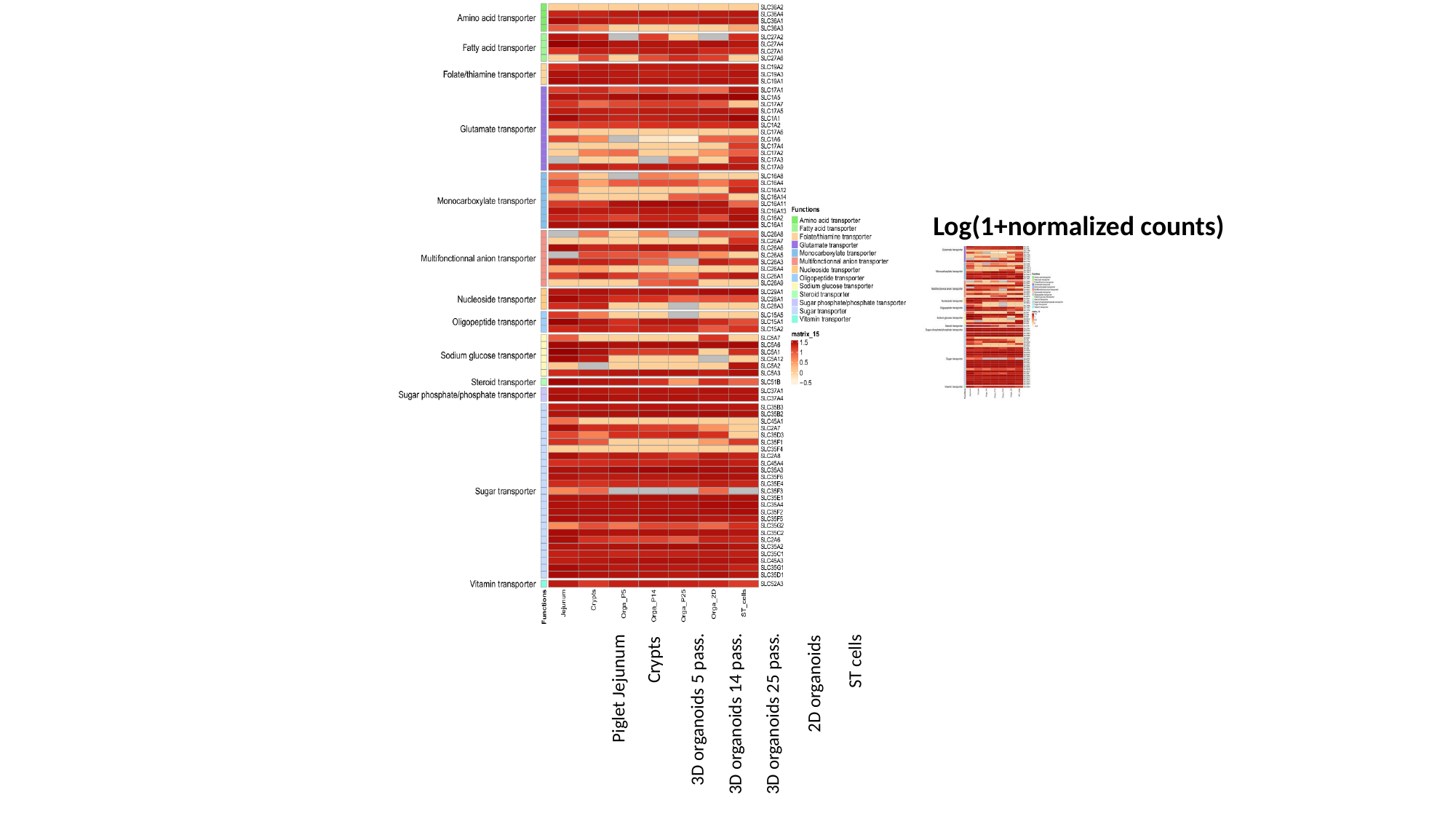

Crypts
ST cells
2D organoids
Piglet Jejunum
3D organoids 5 pass.
Log(1+normalized counts)
3D organoids 14 pass.
3D organoids 25 pass.
